# Environmental dissemination of pathogenic *Listeria monocytogenes* in flowing surface waters in Switzerland

**DOI:** 10.1038/s41598-021-88514-y

**Published:** 2021-04-27

**Authors:** Susanne Raschle, Roger Stephan, Marc J. A. Stevens, Nicole Cernela, Katrin Zurfluh, Francis Muchaamba, Magdalena Nüesch-Inderbinen

**Affiliations:** Institute for Food Safety and Hygiene, Vetsuisse Faculty, University of Zurich, Zurich, Switzerland

**Keywords:** Microbiology, Molecular biology

## Abstract

*Listeria monocytogenes* is an opportunistic pathogen that is widely distributed in the environment. The aquatic environment may represent a potential source for the transmission of *L. monocytogenes* to animals and the food chain. The present study assessed the occurrence of *L. monocytogenes* in 191 surface water samples from rivers, streams and inland canals throughout Switzerland. Twenty-five (13%) of the surface water samples contained *L. monocytogenes*. Whole genome sequence (WGS) data were used to characterize the 25 isolates. The isolates belonged to major lineages I and II, with the majority assigned to either serotype 1/2a (48%), or 4b (44%). The predominant CCs identified were the hypervirulent serotype 4b clones CC1 and CC4, and the serotype CC412; all three have been implicated in listeriosis outbreaks and sporadic cases of human and animal infection worldwide. Two (8%) of the isolates belonged to CC6 which is an emerging hypervirulent clone. All isolates contained intact genes associated with invasion and infection, including *inlA/B* and *prfA*. The four CC4 isolates all harbored *Listeria* pathogenicity island 4 (LIPI-4), which confers hypervirulence. The occurrence of *L. monocytogenes* in river ecosystems may contribute to the dissemination and introduction of clinically highly relevant strains to the food chain.

## Introduction

*Listeria monocytogene*s is a ubiquitous Gram-positive bacterium and an opportunistic pathogen that causes listeriosis in humans and in some animal species^1^. In humans, listeriosis is a potentially lethal infection, with the vulnerable populations such as the elderly, pregnant women, and immunocompromised persons at particular risk for meningitis, sepsis, premature birth, or abortion^2^. In animals, particularly in ruminants, listeriosis can manifest as rhombencephalitis and in its septicemic form may cause abortion, stillbirth, and death^3^.

*Listeria monocytogenes* is classified into four major evolutionary lineages and four PCR based serogroups^4,5^. The majority of *L. monocytogenes* isolates belong to lineage I that is associated with serotypes 1/2b, 3b and 4b, and to lineage II which includes serotype 1/2a, 1/2c, 3a, and 3c^5^. Further, multilocus sequence typing (MLSTs) subdivides these categories into numerous clonal complexes (CCs) and sequence types (STs). Certain serotypes specifically 1/2a, 1/2b and 4b, and certain CCs including hypervirulent strains assigned to CC1, CC4 and CC6, are frequently encountered in clinical cases^5,6^.

Following survival within the gastrointestinal (GI) tract, multiple virulence factors (VFs) enable *L. monocytogenes* to invade and survive within mammalian host cells^7^. Key VFs include InlA and InlB, which belong to a family of internalins that allow the bacteria to incorporate within vacuoles of the host cells, listeriolysin O (LLO) and phospholipases PlcA and PlcB which are involved in vacuolar lysis, as well as ActA, which provides motility within the cytosol^1,8,9^.

*Listeria monocytogenes* virulence genes are mostly organized in clusters located throughout the chromosome, such as the internalin gene operon, several listerial pathogenicity islands (LIPI-1to LIPI-4), and stress survival islets (SSIs). *L. monocytogenes* harboring LIPI-4 are considered hypervirulent and are associated with enhanced invasion and neural and placental infection^6^. By contrast, *L. monocytogenes* isolated from food processing environments and from food are frequently associated with reduced pathogenicity due to truncated and non-functional major virulence factors such as *inlA/B*^10,11^. Such InlA truncations are partially accountable for hypovirulence in *L. monocytogenes* CC9 and CC121, which are major CCs associated with a food origin^6^.

Consumption of food, notably ready-to-eat food, fresh raw produce, and animal-derived food products that are consumed raw is the primary route of exposure of humans to pathogenic *L. monocytogenes *^12^. The occurrence and persistence of *Listeria* in processing plants are frequently caused by environmental recontamination at plant or farm level^13^. At farm level, the route of *L. monocytogenes* infection in ruminants is understood to be contaminated silage and contamination of farm environments including cattle bedding and water throughs^14^. The introduction sources of *L. monocytogenes* to farm animals, and the pathways permitting pathogenic *L. monocytogenes* entering the food chain are currently not completely understood. *Listeria* is widely distributed in the natural environment, with soil representing a key niche for the persistence of globally distributed *L. monocytogenes* isolates^13^. Soil runoff may contaminate water sources which then serve as reservoirs that transfer *Listeria* through the environment^15^. In addition, contaminated sewage or wastewater effluent is reportedly an important cause of *Listeria* in rivers^16,17^, with *L. monocytogenes* found more commonly than other *Listeria* species in surface waters in urban environments^18^. The aquatic ecosystem therefore provides an ideal setting for the circulation of *L. monocytogenes* between the habitats such as soil, plants, animals, natural and urban environments^19^. A number of studies have shown that *Listeria* occurs in waterways in farm environments and agricultural areas, including water which could be used for irrigation^20,21^. Several studies have reported the prevalence of *L. monocytogenes* in river water in the US and Canada, with serogrouping and pulsed field gel electrophoresis (PFGE) analysis revealing serogroups and pulsotypes that were similar to human *L. monocytogenes* isolates^16,21^. Furthermore, surface water strains may carry functional *inlA* genes thus being potentially virulent^15,17^. Although it is therefore recognized that pathogenic *L. monocytogenes* occur in surface water, whole genome sequencing-based information regarding the molecular diversity of *L. monocytogenes* occurring within the aquatic environment is currently limited.

This study was designed to evaluate the occurrence of *L. monocytogenes* in flowing surface waters throughout Switzerland and to characterize the isolated strains using whole genome analyses. We aimed to identify in the aquatic environment, any epidemic clones associated with human and animal infections in order to assess the relevance of such strains to public health as well as the health of animals. Further emphasis was placed on identifying virulence factors (VFs).

## Results

### Occurrence of *L. monocytogenes* in flowing surface water

**Figure 1:**
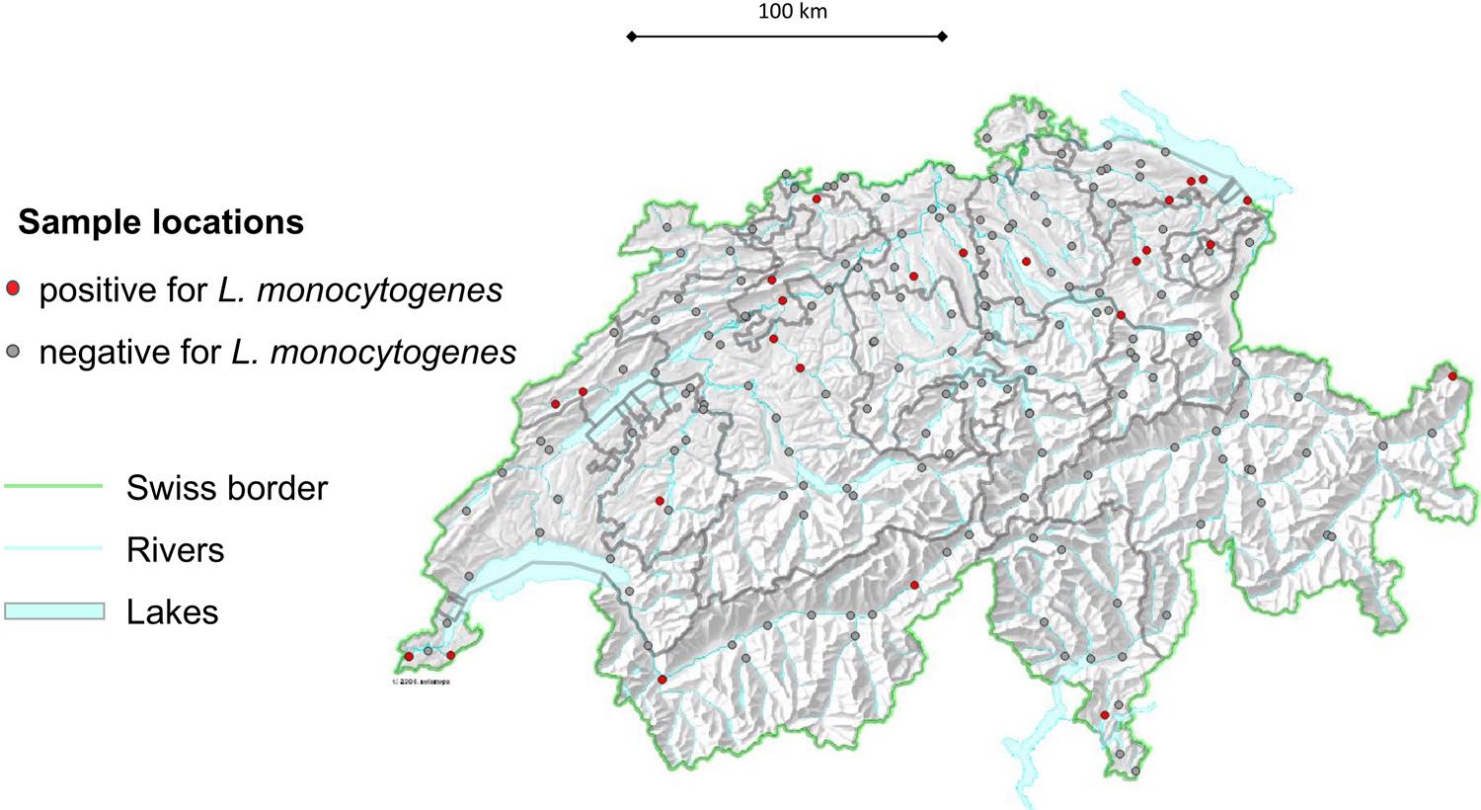
Map of Switzerland showing surface waters, sampling locations, and sites of strain isolation. The map was created using the open source geographic information system (GIS) software QGIS version 3.10 (https://qgis.org).

A total of 191 water samples were collected and analyzed. The geographical distribution of the 191 sampling sites is depicted in Fig. 1. Of the 190 sites, 141 (74%) were located downstream of a wastewater treatment plant (WWTP) (see Supplementary Table S1).

After enrichment, 25 (13%) samples revealed one to three presumptive colonies on OCLA plates. From each plate, one colony was selected for species identification and further analysis using whole genome sequencing (WGS). The positive samples were from sites situated between 280 and 1560 m above sea level, and 19 (76%) were located downstream of WWTPs (Table 1).

**Table 1:** Key features of 25 surface water sampling sites testing positive for *Listeria monocytogenes.*

Water sample ID	Sampling date (dd.mm.yy)	Location (DMS)	Weather conditions (temperature)	Altitude (m)	Type of water body	Downstream of WWTP
L_12	16.01.20	47° 19′ 42″ 8° 36′ 54″	Fair (−2 to 8 °C)	580	Stream	+
L_28	27.01.20	47° 30′ 56″ 7° 43′ 14″	Cloudy (1–3 °C)	290	River	+
L_36	27.01.20	47° 16′ 49″ 7° 31′ 41″	Cloudy (1–3 °C)	680	Stream	+
L_41	11.02.20	47° 33′ 6″ 9° 19′ 34″	Heavy rain (− 1 to 5 °C)	420	Stream	+
L_42	11.02.20	47° 29′ 56″ 9° 13′ 51″	Heavy rain (− 1 to 5 °C)	470	River	+
L_44	11.02.20	47° 19′ 25″ 9° 5′ 6″	Heavy rain (− 1 to 5 °C)	600	Stream	−
L_49	11.02.20	47° 29′ 31″ 9° 33′ 56″	Heavy rain (− 1 to 5 °c)	400	River	+
L_50	11.02.20	47° 21′ 16′ 9° 7′ 45″	Heavy rain (− 1 to 5 °c)	630	River	+
L_51	11.02.20	47° 22′ 0′ 9° 24′ 9″	Heavy rain (− 1 to 5 °c)	800	Stream	+
L_52	11.02.20	47° 33′ 23″ 9° 22′ 42″	Heavy rain (− 1 to 5 °c)	400	River	+
L_57	17.02.20	47° 6′ 35″ 7° 32′ 8″	Cloudy (6–12 °c)	480	Stream	+
L_58	17.02.20	47° 1′ 28″ 7° 38′ 52″	Cloudy (6–12 °c)	560	River	+
L_72	24.02.20	47° 17′ 21″ 8° 8′ 1″	Cloudy (12–15° c)	490	Stream	+
L_86	02.03.20	47° 13′ 14″ 7° 34′ 25″	Cloudy (2–8 °c)	420	River	+
L105	09.03.20	47° 21′ 21″ 8° 20′ 45″	Rain (4–7 °c)	370	River	+
L111	09.03.20	47° 10′ 3″ 9° 0′ 51″	Rain (4–7 °c)	430	Inland canal	+
L124	15.03.20	46° 57′ 43″ 10° 24′ 53″	Fair (2–13 °c)	1560	Stream	−
L127	16.03.20	46° 57′ 14″ 6° 43′ 35″	Fair (− 1 to 10 °c)	730	Stream	+
L128	16.03.20	46° 54′ 60″ 6° 36′ 39″	Fair (− 1 to 10 °c)	730	River	+
L137	16.03.20	46° 10′ 57″ 6° 0′ 36″	Fair (− 1 to 16 °c)	350	River	−
L138	16.03.20	46° 10′ 59″ 6° 11′ 1″	Fair (− 1 to 16 °c)	400	Stream	−
L164	03.05.20	46° 0′ 23″ 8° 54′ 48″	Fair (6–20 °c)	280	River	+
L174	12.05.20	46° 38′ 14″ 7° 3′ 20″	Light rain (3–6 °c)	730	Stream	−
L180	12.05.20	46° 7′ 7″ 7° 4′ 5″	Light rain (3–6 °c)	460	River	−
L188	12.05.20	46° 23′ 30″ 8° 7′ 32″	Cloudy (5–13 °c)	990	River	+

DMS, degrees, minutes, and seconds; WWTP, Wastewater treatment plant.

### Assignment of *L. monocytogenes* lineages and serotypes

After implementation of WGS for the 25 isolates, in silico analyses including serotyping identified thirteen (52%) strains belonging to lineage I, including 11 strains representing serotype 4b, and two belonging to serotype 1/2b (Table 2). The remaining 12 (48%) strains belonged to lineage II and serotype 1/2a (Table 2). The general features of the 25 *Listeria monocytogenes* draft genomes are listed in Table 3.

**Table 2:** Key features of 25 *Listeria monocytogenes* isolates from flowing surface water and of five reference strains.

Strain ID	Location of isolation (DMS)	Date of isolation (dd.mm.yy)	Lineage	Serotype	CC	ST	cgMLST CT	*inlA/B*	LIPI-1	LIPI-3	LIPI-4	SSI-1	GenBank accession no
L28	47° 30′ 56″, 7° 43′ 14″,	27.01.20	I	4b	1	1	14,372	**+**	**+**	**+**	**–**	**–**	JACOFA000000000
L44	47° 19′ 25″, 9° 5′ 6″	11.02.20	I	4b	1	1	14,376	**+**	**+**	**+**	**–**	**–**	JACOEW000000000
L42	47° 29′ 56″, 9° 13′ 51″	11.02.20	I	4b	1	1	14,361	**+**	**+**	**+**	**–**	**–**	JACOEX000000000
L128-3	46° 54′ 60″, 6° 36′ 39″	16.03.20	I	4b	1	1	14,359	**+**	**+**	**+**	**–**	**–**	JACOFH000000000
L174	46° 38′ 14″, 7° 3′ 20″,	12.05.20	I	4b	1	1	14,370	**+**	**+**	**+**	**–**	**–**	JACOFD000000000
L49	47° 29′ 31″, 9° 33′ 56″	11.02.20	I	4b	4	4	14,365	**+**	**+**	**+**	**+**	**–**	JACOEV000000000
L58	47° 1′ 28″, 7° 38′ 52″	17.02.20	I	4b	4	4	14,362	**+**	**+**	**+**	**+**	**–**	JACOEQ000000000
L105	47° 21′ 21″, 8° 20′ 45″	09.03.20	I	4b	4	4	14,364	**+**	**+**	**+**	**+**	**–**	JACOFM000000000
L124	46° 57′ 43″, 10° 24′ 53″	15.03.20	I	4b	4	4	14,366	**+**	**+**	**+**	**+**	**–**	JACOFJ000000000
L12	47° 19′ 42″, 8° 36′ 54″	16.01.20	I	4b	6	6	14,375	**+**	**+**	**+**	**–**	**–**	JACOFK000000000
L164	46° 0′ 23″, 8° 54′ 48″	03.05.20	I	4b	6	6	14,377	**+**	**+**	**+**	**–**	**–**	JACOFE000000000
L52	47° 33′ 23″, 9° 22′ 42″	11.02.20	I	1/2b	224	2332	14,315	**+**	**+**	**+**	**–**	**+**	JACOES000000000
L41	47° 33′ 6″, 9° 19′ 34″	11.02.20	I	1/2b	59	59	14,363	**+**	**+**	**–**	**–**	**–**	JACOEY000000000
L50	47° 21′ 16′ , 9° 7′ 45″	11.02.20	II	1/2a	7	7	14,367	**+**	**+**	**–**	**–**	**+**	JACOEU000000000
L72	47° 17′ 21″, 8° 8′ 1″	24.02.20	II	1/2a	7	7	14,378	**+**	**+**	**–**	**–**	**+**	JACOEP000000000
L36	47° 16′ 49″, 7° 31′ 41″	27.01.20	II	1/2a	11	451	14,371	**+**	**+**	**–**	**–**	**–**	JACOEZ000000000
L86	47° 13′ 14″ 7° 34′ 25″	02.03.20	II	1/2a	14	91	14,314	**+**	**+**	**–**	**–**	**–**	JACOEO000000000
L137	46° 10′ 57″, 6° 0′ 36″	16.03.20	II	1/2a	29	29	14,368	**+**	**+**	**–**	**–**	**–**	JACOFG000000000
L180	46° 7′ 7″, 7° 4′ 5″	12.05.20	II	1/2a	29	29	8916	**+**	**+**	**–**	**–**	**–**	JACOFC000000000
L51	47° 22′ 0′ , 9° 24′ 9″	11.02.20	II	1/2a	37	37	14,374	**+**	**+**	**–**	**–**	**–**	JACOET000000000
L138	46° 10′ 59″ 6° 11′ 1″	16.03.20	II	1/2a	37	37	7538	**+**	**+**	**–**	**–**	**–**	JACOFF000000000
L57	47° 6′ 35″, 7° 32′ 8″	17.02.20	II	1/2a	412	412	14,369	**+**	**+**	**–**	**–**	**–**	JACOER000000000
L111	47° 10′ 3″, 9° 0′ 51″	09.03.20	II	1/2a	412	412	14,360	**+**	**+**	**–**	**–**	**–**	JACOFL000000000
L127	46° 57′ 14″, 6° 43′ 35″	16.03.20	II	1/2a	412	412	14,373	**+**	**+**	**–**	**–**	**–**	JACOFI000000000
L188	46° 23′ 30″, 8° 7′ 32″	12.05.20	II	1/2a	412	412	13,675	**+**	**+**	**–**	**–**	**–**	JACOFB000000000
LL195^a^	n.a	n.a	I	4b	1	1	24	**+**	**+**	**+**	*** − ***	*** − ***	HF558398
N2306^a^	n.a	n.a	I	4b	4	4	2506	**+**	**+**	**+**	**+**	*** − ***	CP011004
EGD-e^a^	n.a	n.a	II	1/2a	9	35	1	**+**	**+**	*** − ***	*** − ***	**+**	NC003210
N1546^a^	n.a	n.a	II	1/2a	8	8	3614	**+**	**+**	*** − ***	*** − ***	**+**	CP013724
N12-1273^a^	n.a	n.a	II	1/2a	412	412	9302	**+**	**+**	*** − ***	*** − ***	*** − ***	QYFZ00000000

^a^ reference strains; CC, clonal complex; CT, cluster type; cgMLST, core genome multilocus sequence type; *inlA/B*, full length internalin genes A and B; LIPI, *Listeria.*

*monocytogenes* pathogenicity island; n.a., not applicable; SSI, stress survival islet; ST, sequence type; +, presence of gene(s); –, absence of gene(s).

**Table 3:** General features of the 25 *Listeria monocytogenes* draft genomes and number of comparable genomes available in the NENT database^a^.

Strain ID	No. core genes	No. accessory genes	No. unique genes	No. exclusively absent genes	N50	L50	ST^b^	No. genomes in NENT-DB
L28	2497	306	14	1	476,849	3	1	30
L44	2497	357	25	1	476,856	3	1	30
L42	2497	267	56	2	479,292	3	1	30
L128-3	2497	365	60	7	524,722	3	1	30
L174	2497	327	0	1	476,855	3	1	30
L49	2497	264	0	2	478,300	3	4	12
L58	2497	265	0	1	478,305	3	4	12
L105	2497	305	13	4	478,306	3	4	12
L124	2497	328	3	2	541,335	2	4	12
L12	2497	338	3	0	551,226	3	6	56
L164	2497	271	0	5	510,038	2	6	56
L52	2497	291	21	3	476,821	3	2332	0
L41	2497	294	37	3	478,264	3	59	4
L50	2497	246	32	0	609,560	2	7	9
L72	2497	379	10	2	446,908	3	7	9
L36	2497	299	13	3	571,154	2	451	1
L86	2497	352	111	4	582,512	2	91	1
L137	2497	295	0	0	1,490,250	1	29	1
L180	2497	331	43	1	1,489,724	1	29	1
L51	2497	321	0	0	1,497,622	1	37	8
L138	2497	354	12	1	1,530,524	1	37	8
L57	2497	275	0	2	543,302	2	412	4
L111	2497	277	0	0	543,302	2	412	4
L127	2497	276	0	0	526,717	2	412	4
L188	2497	277	0	0	543,302	2	412	4

^a^ database of the Swiss National Reference Centre for Enteropathogenic Bacteria and *Listeria* (NENT).

^b^ST, sequence type; strains were assigned and grouped into STs in accordance with the BIGSdb-*L. monocytogenes* platform. For details see main text.

### Identification of epidemic and outbreak clones

To assess the clinical relevance of *L. monocytogenes* occurring in the aquatic environment, the genomes of the 25 strains were subjected to detailed in silico analysis. Based on the seven-gene MLST scheme provided by the BIGSdb-*L. monocytogenes* platform (https://bigsdb.pasteur.fr/listeria), a total of 11 STs which corresponded to 11 CCs were identified (Table 2). The predominant CCs were CC1 (20%), CC4 (16%), and CC412 (16%) (Table 2). *L. monocytogenes* serotype 4b CC1 and CC4 are epidemic clones associated with human listeriosis outbreaks worldwide, and both clones are also frequent among strains causing listeriosis in ruminants^6,22,23^. By contrast, *L. monocytogenes* serotype 1/2a CC412 is only sporadically associated with human infections but is prevalent among strains causing rhombencephalitis in cattle^6,22^. Two further strains (8%) were assigned to CC6, which is an emerging epidemic *L. monocytogenes* serotype 4b clone causing major outbreaks and severe forms of human listerial meningitis worldwide^6,24^. Other CCs identified among the strains in this study included *L. monocytogenes* serotype 1/2a CC7, CC11 and CC29, all of which have been associated with outbreaks occurring in the US between 1987 and 2011, and further found to represent environmental strains that persist within food production and livestock environments globally^23,25–27^. On the other hand, *L. monocytogenes* serotype 1/2a CC37 found in two samples in this study, is associated with abortion in small ruminants and cattle and found frequently in wildlife and ruminant environments^13,25,28^. Further, *L. monocytogenes* CC37 is prominent in milk samples from US dairy farms^29^.

### Analysis of strain relatedness

**Figure 2:**
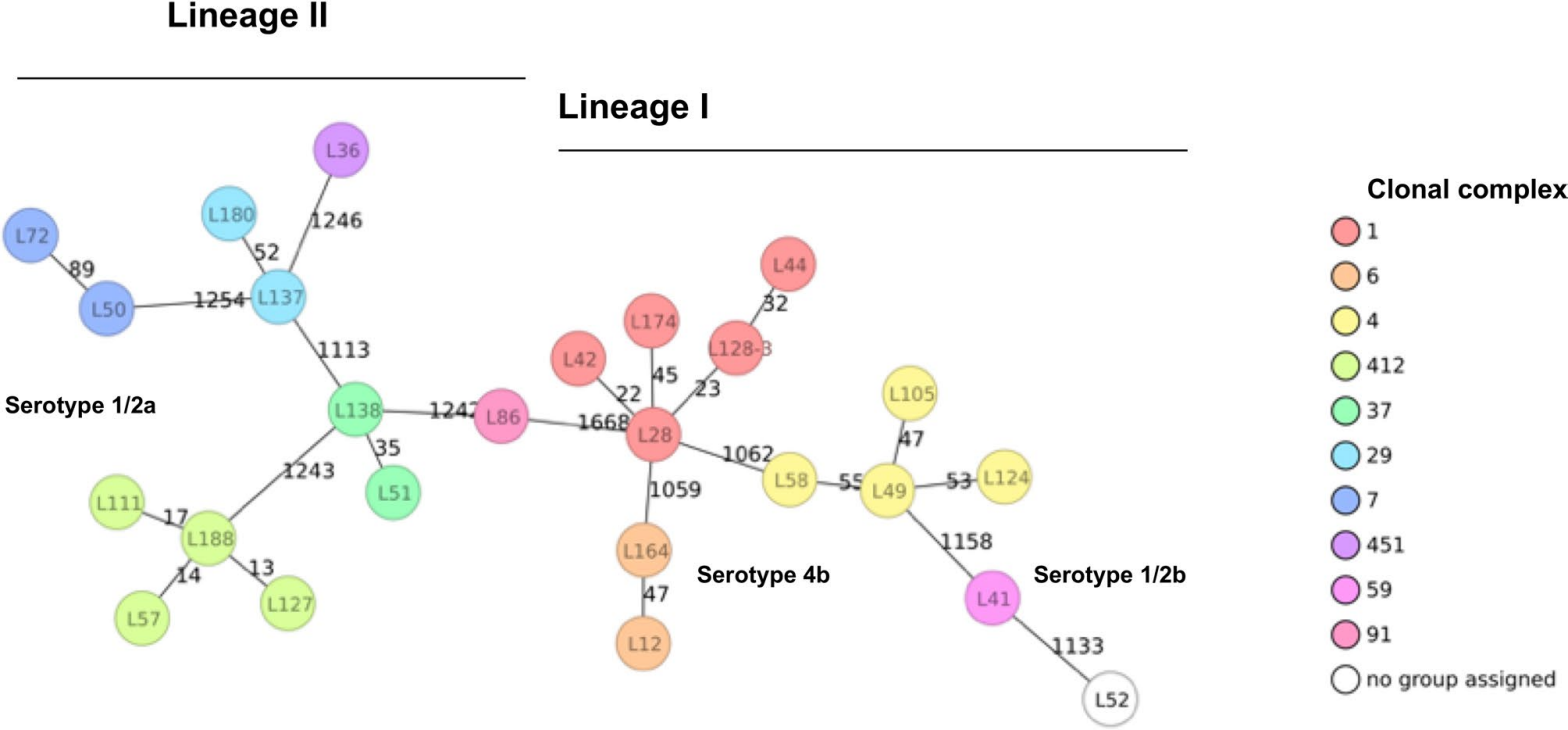
Minimum-spanning tree based on cgMLST allelic profiles of 25 *Listeria monocytogenes* isolated from surface waters. Each circle represents an allelic profile based on sequence analysis of 1,701 cgMLST target genes. The numbers on connecting lines represent the number of allelic differences between two strains. Each circle contains the strain ID, and CCs are color coded. Lineages and serotypes are indicated.

**Figure 3:**
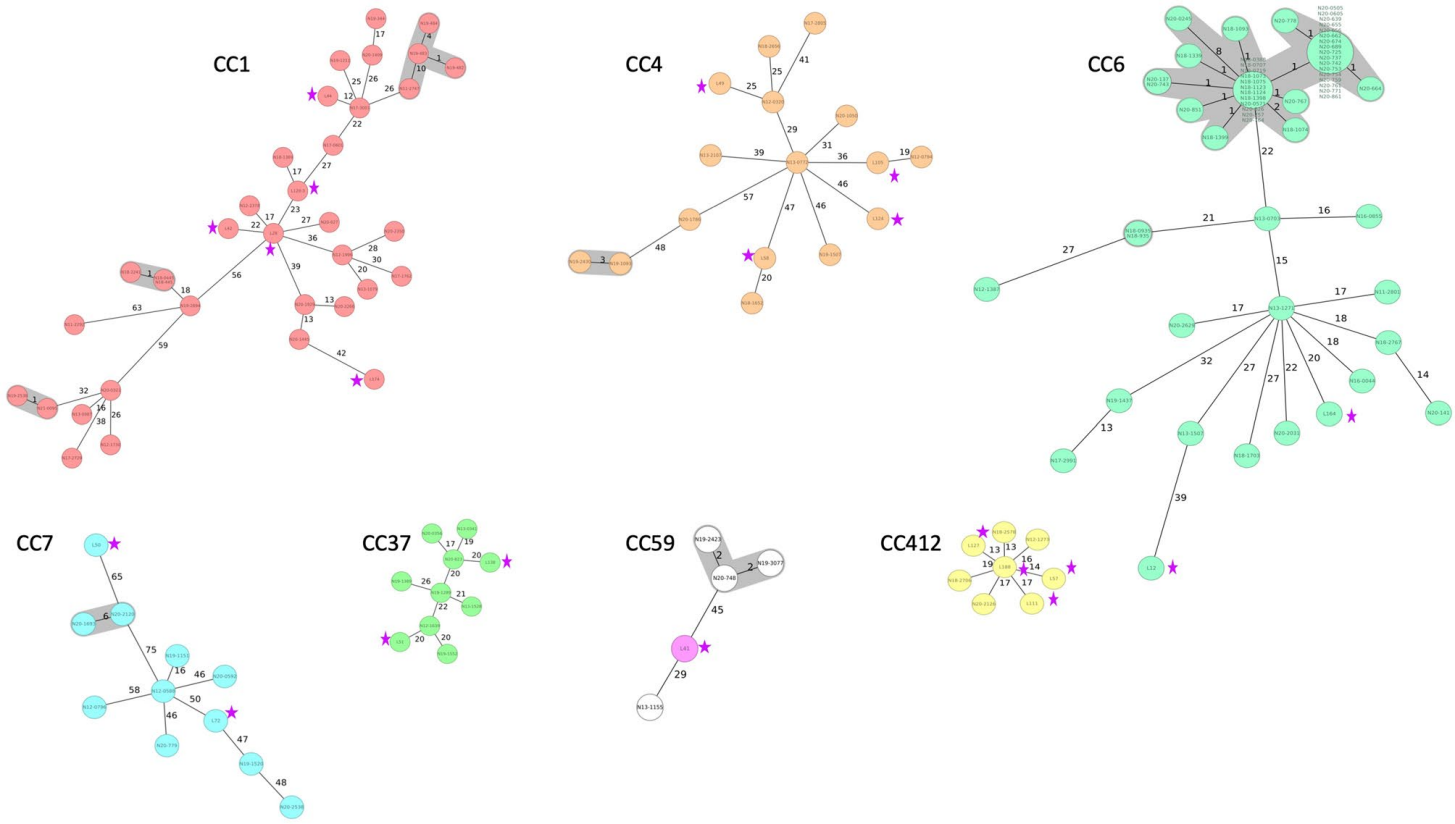
Minimum-spanning trees based on cgMLST allelic profiles of 25 *Listeria monocytogenes* isolated from surface water and 368 genome-sequenced clinical *L. monocytogenes* isolated during 2011–2020 available in the database of the Swiss National Reference Centre for Enteropathogenic Bacteria and *Listeria* (NENT) in Switzerland. Strains are grouped by clonal complex (CC). Each circle represents an allelic profile based on sequence analysis of 1,701 cgMLST target genes. The numbers on connecting lines represent the number of allelic differences between two strains. Clusters were defined as isolates containing ≤ 10 different alleles between a pair of isolates. Strains from this study are indicated with a pink star.

The 25 *L. monocytogenes* strains belonged to 25 different cgMLST types (CTs), as shown in Table 2. The population structure of the strains was visualized by constructing a phylogenetic tree based on cgMLST. The isolates grouped according to lineages and serotypes, but were phylogenetically clearly distinct from each other, with ≥ 10 different alleles between each pair of neighboring isolates (Fig. 2). The genomes of the CC1, CC4, CC6, CC7, CC37, CC59, and CC412 isolates were compared with the available genomes of corresponding CCs present in the database of the Swiss National Reference Centre for Enteropathogenic Bacteria and *Listeria* (NENT) which collects all *L. monocytogenes* strains from confirmed human listeriosis cases nationwide and performs Illumina-based whole genome sequencing. The cgMLST-based phylogenetic trees are shown in Fig. 3 and the number of *L. monocytogenes* genomes in the NENT database are listed in Table 3. None of the 25 strains from this study clustered with a strain in the database, thereby ruling out a direct match with any *L. monocytogenes* reported from a case of human disease in Switzerland.

### Assessment of virulence attributes

Pan-genomic analysis was performed to identify the presence of virulence genes associated with invasion of mammalian host cells, hypervirulence, or neural and placental infections.

Internalin A, encoded by *inlA* is a key factor associated with pathogenic *L. monocytogenes*. Premature stop codons within *inlA*, often observed in food-associated and environmental strains, result in the presence of truncated InlA and reduced or loss of virulence^30^. In this study, all isolates contained full length *inlA* genes and were therefore potentially virulent. Among the 25 strains, there were several lineage and clonal complex specific SNP-introduced amino acid substitutions in *inlA* compared to the reference strain EDG-e. For example, the amino acid substitution R3K was only detected in strains belonging to lineage I, whilst Y774D was only detected in *L. monocytogenes* CC4 and CC6 (see Supplementary Table S2).

Further, *L. monocytogenes* CC6 (strains L12 and L164, respectively) all had a characteristic triple amino acid deletion in the pre-anchor region of InlA, the impact of which is not yet known^30^ (see Supplementary Table S2).

In addition to *inlA*, the *L. monocytogenes* genomes in this study showed a variable combination of other internalin genes due to the presence or absence of *inlG* and *inlL* (see Supplementary Fig. S2). The *inlG* gene was lacking in lineage I strains, with the exception of two CC6 strains (L12 and L164). Similarly, *inlL* was absent in all lineage I strains as well as in three lineage II strains (L36, L86, and L180) (Supplementary Table 2 and Supplementary Fig. S2).

LIPI-1 is a PrfA dependent virulence gene cluster consisting of six genes (*prfA, plcA, hly, mpl, actA* and *plcB*) that are crucial for the infection cycle of *L. monocytogenes*^31^*.* In this study, LIPI-1was present in all 25 strains (Supplementary Table 2 and Supplementary Fig. S2). All strains contained full length *prfA* genes (see Table S3). There were lineage and clonal complex specific SNP-introduced amino acid substitutions in *hly*, for example L35S and I438V were only detected in lineage I strains whilst V433I was unique to CC412 strains (see Supplementary Table S4).

There were various other minor lineage and strain specific genetic differences detected in further virulence factors including some amino acid changing SNPs in *plcA*, *plcB* and *actA*, but none that have previously been reported to alter functions of these proteins.

LIPI-3 encodes a biosynthetic cluster involved in the production of listeriolysin S (LLS), a hemolytic and cytotoxic factor postulated to play a role in *Listeria* gastrointestinal colonization^32^. LIPI-3 is strongly associated with lineage I strains and was present in all lineage I strains of this study with the exception of strain L41 which belonged to serotype 1/2b ST59 (Supplementary Table 2 and Supplementary Fig. S2).

LIPI-4 contains six genes encoding cellobiose-type phosphotransferase systems (PTS) that can enhance invasion, leading to neural and placental listeriosis^6^. The presence of LIPI-4 is strongly associated with hypervirulence of CC4 strains. Accordingly, LIPI-4 was identified among all CC4 strains in this study (Table 2 and Fig. S2).

SSI-1 is a five gene islet that contributes to the growth of *L. monocytogenes* under suboptimal conditions^33,34^. This islet has been previously shown to be a feature of *L. monocytogenes* CC7 and CC8 associated with persistence in food-processing plants but is also found in sporadic environmental strains^26^. In this study, SSI-1 was identified only in strains L50 and L72 both (CC7), and in L52 belonging to an ST2332 (Supplementary Table 2 and Supplementary Fig. S2).

Other VF including genes involved in adherence, intracellular survival, regulation of transcription and translation and surface protein anchoring were present in most of the strains, with some notable lineage, clonal complex, and serotype associated differences (see Supplementary Fig. S2). For instance, the adherence gene *ami*^35^ was lacking in lineage I except in strain L41. Further, strains belonging to serotype 4b all contained *aut_IVb* which is an allele of the invasion gene *aut *^36^, and all lacked *tagB*, a gene involved in teichoic acid biosynthesis^37^. All serotype 1/2a and 1/2b strains were as expected without the genes *gltA* and *gltB* which are serotype 4b-specific genes involved in teichoic acid biosynthesis^38^. Finally, all *L. monocytogenes* CC412 lacked the adherence gene *lapB *^35^.

### Identification of antimicrobial, heavy metal and disinfectant resistance determinants

All 25 *L. monocytogenes* strains from this study contained four intrinsic antibiotic resistance genes, including the fosfomycin hydrolase gene *fosX*, the antibiotic efflux pump gene *lin*, the quinolone resistance efflux pump gene *norB*, and the sulfonamide resistance gene *sul*. Cadmium resistance genes *cadA1* and *cadC1* were detected in strain L86 (CC14). No arsenic or benzalkonium chloride resistance genes were detected (see Supplementary Fig. S2).

## Discussion

In this nationwide study, we recovered *L. monocytogenes* from water samples from rivers and streams localized within agricultural and nonagricultural environments, urbanized areas, and mountainous regions up to altitudes of 1560 m above sea level. The majority (21 /84%) of the positive samples was retrieved within the geographical region of Switzerland that belongs to the so-called Central Plain. This area is characterized by settlement and urban areas, as well as agricultural areas, and represents the most densely populated region of Switzerland^39^. Further, with 76% of the positive samples located in the proximity to WWTP, anthropogenic sources of *L. monocytogenes* strains retrieved in this study appear likely, however, this observation needs to be confirmed by additional investigations that include further data collection. Indeed, previous studies suggest that *Listeria* species survive conventional wastewater treatment processes and that effluents of WWTPs are potential sources of clinically important *L. monocytogenes *^40,41^. However, the prevalence and diversities of the *L. monocytogenes* strains may have been influenced by the recovery procedure. In our study, the methodology included enrichment in HFB, as opposed to previous studies using other protocols to isolate *Listeria* from water samples, such as Universal Pre-enrichment Broth (UPB)^21^, or selective Listeria Enrichment Broth (LEB)^15,17^. It cannot be excluded that some lineages or serotypes of *L. monocytogenes* may differ in their ability to recover in HFB, and caution should be applied when comparing results obtained using different methodologies.

The prevalence of *L. monocytogenes* in surface water in this study was 13%. By comparison, *L. monocytogenes* was identified in 10% of surface water sampled in Canada^15^, in 13% in New York State^42^, 31% in Mid-Atlantic US^21^. Notably, *L. monocytogenes* was not detected in surface water in Austria^13^. However, prevalence of *L. monocytogenes* in natural water bodies may vary according to recovery methodology and sampling season^20,21^. Therefore, the lack of periodic sampling and the lack data for the summer and autumn seasons in this study could have influenced the results and it cannot be excluded that extending the study period to include warm seasons may have had an impact on prevalence and variety of the *L. monocytogenes* present in flowing surface waters. Therefore, our results may not apply directly to *L. monocytogenes* recovered from water during warm seasons. Nevertheless, the data from this study highlights the broad geographical distribution of clinically relevant *L. monocytogenes* in the aquatic ecosystem.

Among the isolates, the majority belonged to serotypes and clonal complexes corresponding to those from human listeriosis outbreaks and sporadic cases of human and animal infection. In our study, the majority of the strains were either serotype 1/2a (48%), or 4b (44%). These results are similar to those reported for surface waters analyzed in Canada, where serotypes 1/2a/3a and 4b/4d/4e constituted 49% and 32% of the isolates, respectively^15^. By contrast, other investigations found serotype 1/2a among 67% of water-derived *L. monocytogenes* in a further Canadian study, while *L. monocytogenes* recovered from water samples in California belonged predominantly to serotype 4b/4d/4e^16^. In spite of the limited number of isolates in the present study and the differences in methodologies and study settings used by previous investigators, these results of these earlier studies are supportive of our observation that *L. monocytogenes* populations in the aquatic environment contain serotypes that may cause listeriosis. Further, virulence gene profiling revealed that all the strains harbored intact genes associated with invasion and infection, underlining the virulence potential and clinical relevance of *L. monocytogenes* from the aquatic environment. These findings, although based on the analysis of a small number of isolates, are different from data on VFs found in many but not all isolates of serotypes 1/2a, 1/2b and 1/2c from food and food processing environments for example in Ireland and in the US, where *inlA* is truncated in 31% and in 45% of the isolates, respectively^10,43^. Our data are also supportive of a previous report regarding the integrity of the virulence gene *inlA* in *L. monocytogenes* recovered from natural waters^17^. Notably, there was a lack of benzalkonium and arsenic resistance genes and a very low prevalence of cadmium resistance genes among the strains in this study. These determinants are frequently associated with *L. monocytogenes* isolated from food and from humans, and their scarcity among the isolates in this study may reflect specific adaptations in the natural environment, consistent with a recent study that observed a very low prevalence of cadmium resistance genes among *L. monocytogenes* from wildlife^44^.

A considerable proportion (16%) of the clones belonged to CC4 which contains LIPI-4 and is considered hypervirulent based on its neurovirulence and capacity for placental infection^6^. CC4 is highly associated with human isolates and, in contrast to other serotype 4b strains, to our knowledge has not been described in surface water so far^45^.

Likewise, CC1 and CC6 are major contributors to human listeriosis, and recent years have seen an increase of severe listeriosis cases related to *L. monocytogenes* CC6, a clone which was first implicated in a multistate outbreak in the US in 1998–1999^46^, and has since disseminated globally, causing one of the world’s largest listeriosis outbreaks in South Africa in 2017–2018, a large outbreak in Germany during 2018–2019, a local outbreak in Switzerland in 2016^47–49^ , and very recently, a nationwide outbreak in Switzerland in 2018–2020 that caused 34 cases and 10 deaths^50^.

The occurrence in surface water of *L. monocytogenes* belonging to CCs associated with disease highlights the potential of rivers, streams and inland canals as a reservoir for pathogenic *L. monocytogenes*. In particular, the use of river water for crop irrigation during dry seasons may allow *L. monocytogenes* in the water to enter the food chain. Irrigation has repeatedly been associated with an increased risk of pre-harvest produce contamination by *L. monocytogenes,* particularly if the irrigation water is drawn from surface water^19,20,51,52^. Further, irrigation within three days of harvest is associated with *L. monocytogenes* in produce production environments^42^. Therefore, river sourced irrigation water may indicate a public health risk, should contaminated product be consumed raw.

Similarly, the surface water samples in this study contained particular CCs that have reportedly caused bovine listeriosis, with *L. monocytogenes* CC1, CC4, and CC412 among the most common causes of ruminant encephalitis in central Europe^28^. These CCs are also frequently detected in the cattle farm environment including feed, drinking troughs, ruminant feces and manure^25,28^^.^ However, the introduction routes of these CCs to the farm environment are currently not well understood, although spoiled silage is considered to be the primary source^3,14^. The occurrence of *L. monocytogenes* in rivers reported in this study points towards surface water a possible further source of clones causing disease in cattle. Notably, the use of river water for watering cattle may represent a possible exposure to bovine pathogenic *L. monocytogenes*.

## Conclusions

This study demonstrates that *L. monocytogenes* circulating in the aquatic environment belong to CCs and contain the same virulence traits as *L. monocytogenes* that are frequently isolated from human and animal clinical cases and from globally occurring outbreaks, including hypervirulent clones CC1, CC4, and CC6.

Our data contribute to a better understanding of the diversity of *L. monocytogenes* present in flowing surface waters. The results may provide information to improve crop irrigation strategies and cattle watering practices that aim to reduce the transmission of foodborne pathogens from surface water to fresh produce and to the farm environment.

## Material and methods

### Sampling

A total of 191 water samples from different rivers and streams and inland canals throughout Switzerland were collected between January and May 2020. Sampling sites were located between 200 m and 1,730 m above sea level (see Supplementary Table S1). Water was taken from large rivers at 1 m depth using collection poles and sterile 500 mL containers. Smaller water bodies were sampled at 0.2–0.3 m depth using sterile 500 mL containers. The water samples were transported to the laboratory in a cool box. Each sample was stored at 4 °C for a maximum of 18 h. At each sampling site, weather conditions, ambient temperature and proximity to wastewater treatment plants were recorded.

### Bacterial isolation

From each water sample 100 ml were passed through sterile, 0.22 μm membrane filters (Millipore, Billerica, MA, USA). For enrichment, the filters were incubated in 50 ml Half Frazer Broth (HFB, BioRad, Cressier, Switzerland) in sterile blender bags (Seward, Worthing, UK) at 30 °C for 48 h. One loopful each of the enriched cultures was streaked on Oxoid chromogenic Listeria agar (OCLA) plates (Oxoid, Pratteln, Switzerland) and incubated under aerobic conditions at 37 °C for 48 h. Presumptive *Listeria* colonies exhibiting green morphologies and opaque halos were subcultured on OCLA plates at 37 °C for 48 h. *L. monocytogenes* isolates were grown on sheep blood agar (Difco Laboratories) at 37 °C for 24 h, and kept at − 80 °C in brain heart infusion (BHI) broth stocks (Oxoid, Hampshire, UK) containing 15% glycerol.

## Reference strains

To confirm lineages and serotypes, an average nucleotide identity (ANI) comparison was performed to create a phylogenetic tree that was calibrated with five *L. monocytogenes* genomes of known lineages and serotypes as reference strains (see Supplementary Fig. S1). The *L. monocytogenes* serotype 4b and 1/2b isolates from this study were compared to previously described outbreak strains LL195 (GenBank accession no. HF558398) and N2306 (CP011004). *L. monocytogenes* LL195 (CC1) was isolated from Swiss Vacherin Mont d’Or cheese during an outbreak 1983–1987^53^, and *L. monocytogenes* N2306 was detected in ready-to-eat salad during an outbreak 2013–2014^54^.

The *L. monocytogenes* serotype 1/2a isolates were compared to the reference strain *L. monocytogenes* EGD-e (NC003210), and to strains N1546 (CP013724) and N12-1273 (QYFZ00000000). *L. monocytogenes* EGD-e (CC9) was originally isolated from a rodent and is a widely used reference strain^55^. *L. monocytogenes* N1546 (CC8) is a clinical isolate recovered during the 2011 outbreak linked to ham products^56,57^. N12-1273 (CC412) is a previously characterized human listeriosis isolate^49^.

### Whole genome sequencing

Genomic DNA was isolated using the DNeasy Blood and Tissue Kit (Qiagen, Hilden, Germany). Sequencing libraries were prepared using the Illumina Nextera DNA preparation kit and sequencing was performed on an Illumina MiSeq sequencer (Illumina, San Diego, CA, USA). Genomes were sequenced with a minimal coverage of 50x. Following a quality assessment with FastQC (http://www.bioinformatics.babraham.ac.uk/projects/fastqc/), the reads were assembled with Shovill 1.0.9 and Spades 3.12.0^58^ and integrated into the Ridom SeqSphere + software version 5.1.0 (Ridom, Münster, Germany)^59^.

### Serotyping and multilocus sequence typing

In silico serotyping was performed in SeqSphere + using gene targets described previously^4^. Classic MLST determination based on seven housekeeping genes were performed in accordance with the *L. monocytogenes* BIGSdb-L. monocytogenes platform (https://bigsdb.pasteur.fr/listeria).

### Core genome MLST and Single-Nucleotide Polymorphism Analysis

Core genome MLST (cgMLST) analysis was performed in accordance to the core genome defined by Ruppitsch et al.^59^. Sequences were blasted against 1701 genes of the reference genome of strain EGD-e, using the standard settings^59^. Cluster types (CTs) were determined upon submission to the *L. monocytogenes* cgMLST Ridom SeqSphere + server (http://www.cgmlst.org/ncs/schema/690488/). Missing genes were ignored in all samples. Minimum spanning trees (MSTs) were generated in Ridom SeqSphere + version 5.1.0 for visualization of strain relatedness. Clusters were defined as isolates containing ≤ 10 different alleles between a pair of isolates^59^.

Single-nucleotide polymorphism (SNP) analysis was performed using tparsnp in the harvest suite with option -c ignore MUMi activated^60^. An ANI was calculated based on MUMmer alignments as described previously^61^. Each isolate was compared to a reference strain of the same serotype, as listed above.

### Pan- and Core Genome Profiling

The Bacterial Isolate Genome Sequence database (BIGSdb)^62^ was used to generate the pan-genome of the 25 surface water isolates together with the five reference strains. The presence or absence of genes, including virulence genes, pathogenicity islands, antimicrobial resistance, heavy metal resistance, and biocide resistance genes across each genome was verified by manual curation using Basic Local Alignment Search Tool (BLAST) version 2.10.1 on a CLC genomics Workbench version 20.0.3.

### Geographical map

Geospatial visualization was carried out by plotting GPS coordinates of the sampling sites onto a geographical map using the open source geographic information system (GIS) software QGIS (https://qgis.org).

## Supplementary Information

Supplementary Information.

## Data Availability

This Whole Genome Shotgun project has been deposited at DDBJ/ENA/GenBank under the accession numbers JACOEO000000000- JACOFM000000000. The versions described in this paper are versions JACOEO010000000- JACOFM010000000. Accession numbers for the individual *L. monocytogenes* strains from this study are listed in Table 2. The BioProject number is PRJNA657153.
