# Supplementary material for: Environmental dissemination of pathogenic Listeria monocytogenes in flowing surface waters in Switzerland
Source: Sci Rep. 2021 Apr 27;11:9066. doi: 10.1038/s41598-021-88514-y (PMC8079687; doi:10.1038/s41598-021-88514-y)
Supplement: Supplementary file 1 — Supplementary Information. [file 41598_2021_88514_MOESM1_ESM.pdf]

**Supplementary Information**

# Environmental dissemination of pathogenic *Listeria monocytogenes* in flowing surface waters in Switzerland

Susanne Raschle,[a] Roger Stephan,[a] Marc J.A. Stevens,[a] Nicole Cernela,[a] Katrin Zurfluh,[a] Francis Muchaamba,[a] Magdalena Nüesch-Inderbinen[a] *

[a]Institute for Food Safety and Hygiene, Vetsuisse Faculty, University of Zurich, Zurich, Switzerland

* Corresponding author: magdalena.nueesch-inderbinen@uzh.

**Table S1:** Geographical features of water samples collected from suface water bodies throughout Switzerland, 2020**.**

| Water sample ID* | Sampling date (dd.mm.yy) | Location of isolation (DMS) | Weather conditions (temperature) | Altitude (m) | Type of water body | Name of water body | Downstream of WWTP** |
|---|---|---|---|---|---|---|---|
| L_1 | 14.01.20 | 47°36`23`` 9°6`40`` | fair (0-5°C) | 470 | stream | Chemibach | + |
| L_2 | 14.01.20 | 47°35`39`` 8°57`55`` | fair (0-5°C) | 400 | stream | Chemebach | + |
| L_3 | 14.01.20 | 47°32`29`` 8°54`27`` | fair (0-5°C) | 430 | river | Murg | + |
| L_4 | 14.01.20 | 47°29`33`` 8°59`0`` | fair (0-5°C) | 510 | river | Murg | + |
| L_5 | 14.01.20 | 47°34`6`` 9°6`23`` | fair (0-5°C) | 430 | river | Giessen | + |
| L_6 | 14.01.20 | 47°38`19`` 9°13`8`` | fair (0-5°C) | 410 | stream | Töbelibach | - |
| L_7 | 14.01.20 | 47°35`18`` 8°56`27`` | fair (0-5°C) | 390 | river | Thur | - |
| L_8 | 16.01.20 | 47°26`27`` 8°42`13`` | fair (-2-8°C) | 480 | river | Kempt | + |
| L_9 | 16.01.20 | 47°22`15`` 8°48`34`` | fair (-2-8°C) | 620 | river | Luppmen | + |
| L_10 | 16.01.20 | 47°15`13`` 8°47`46`` | fair (-2-8°C) | 490 | stream | Klausbach | + |
| L_11 | 16.01.20 | 47°17`45`` 8°43`13`` | fair (-2-8°C) | 450 | stream | Lieburgerbach | + |
| L_12 | 16.01.20 | 47°19`42`` 8°36`54`` | fair (-2-8°C) | 580 | stream | Chliweidlibach | + |
| L_13 | 16.01.20 | 47°31`6`` 8°39`17`` | fair (-2-8°C) | 410 | river | Töss | + |
| L_14 | 20.01.20 | 47°39`42`` 8°58`17`` | fair (-2-1°C) | 400 | stream | Feldbach | + |
| L_15 | 20.01.20 | 47°38`20`` 8°46`25`` | fair (-2-1°C) | 430 | stream | Mülibach | + |
| L_16 | 20.01.20 | 47°45`14`` 8°41`37`` | fair (-2-1°C) | 440 | river | Biber | - |
| L_17 | 20.01.20 | 47°41`18`` 8°27`14`` | fair (-2-1°C) | 410 | inland canal | Klingengraben | + |
| L_18 | 20.01.20 | 47°34`10`` 8°28`47`` | fair (-2-1°C) | 350 | river | Glatt | + |
| L_19 | 20.01.20 | 47°35`57`` 8°17`43`` | fair (-2-1°C) | 320 | river | Rhein | + |
| L_20 | 20.01.20 | 47°29`6`` 8°17`49`` | fair (-2-1°C) | 350 | river | Limmat | - |
| L_21 | 20.01.20 | 47°29`2`` 8°12`52`` | fair (-2-1°C) | 340 | river | Aare | + |
| L_22 | 20.01.20 | 47°27`33`` 8°14`42`` | fair (-2-1°C) | 340 | river | Reuss | + |
| L_23 | 20.01.20 | 47°31`7`` 8°0`55`` | fair (-2-1°C) | 340 | stream | Sissle | + |
| L_24 | 20.01.20 | 47°24`46`` 8°5`4`` | fair (-2-1°C) | 370 | river | Aare | + |
| L_25 | 27.01.20 | 47°34`36`` 7°50`26`` | cloudy (1-3°C) | 290 | stream | Möhlin | + |

| Water sample ID* | Sampling date (dd.mm.yy) | Location of isolation (DMS) | Weather conditions (temperature) | Altitude (m) | Type of water body | Name of water body | Downstream of WWTP** |
|---|---|---|---|---|---|---|---|
| L_26 | 27.01.20 | 47°33`11`` 7°47`41`` | cloudy (1-3°C) | 280 | stream | Magdenbach | - |
| L_27 | 27.01.20 | 47°33`5`` 7°45`55`` | cloudy (1-3°C) | 270 | river | Rhein | + |
| L_28 | 27.01.20 | 47°30`56`` 7°43`14`` | cloudy (1-3°C) | 290 | river | Ergolz | + |
| L_29 | 27.01.20 | 47°35`18`` 7°35`22`` | cloudy (1-3°C) | 250 | river | Rhein | + |
| L_30 | 27.01.20 | 47°32`47`` 7°37`23`` | cloudy (1-3°C) | 260 | stream | Birs | + |
| L_31 | 27.01.20 | 47°25`41`` 7°26`42`` | cloudy (1-3°C) | 420 | stream | Lützel | + |
| L_32 | 27.01.20 | 47°21`54`` 7°21`1`` | cloudy (1-3°C) | 410 | stream | La Sorne | - |
| L_33 | 27.01.20 | 47°21`31`` 7°8`19`` | cloudy (1-3°C) | 440 | stream | Risseau du Doubs | + |
| L_34 | 27.01.20 | 47°25`59`` 7°4`49`` | cloudy (1-3°C) | 410 | stream | Làllaine | + |
| L_35 | 27.01.20 | 47°16`42`` 7°23`48`` | cloudy (1-3°C) | 550 | stream | La Raus | - |
| L_36 | 27.01.20 | 47°16`49`` 7°31`41`` | cloudy (1-3°C) | 680 | stream | Dünnern | + |
| L_37 | 27.01.20 | 47°77`26`` 8°27`74`` | cloudy (1-3°C) | 1893 | stream | Tannenbach | - |
| L_38 | 09.01.20 | 46°36`47`` 9°35`21`` | fair (-8-2°C) | 1330 | river | Julia | + |
| L_39 | 09.01.20 | 46°44`34`` 9°25`55`` | fair (-8-2°C) | 630 | river | Hinterrhein | + |
| L_40 | 09.01.20 | 46°49`29`` 9°24`26`` | fair (-8-2°C) | 580 | river | Vorderrhein | - |
| L_41 | 11.02.20 | 47°33`6`` 9°19`34`` | heavy rain (-1-5°C) | 420 | stream | Aach | + |
| L_42 | 11.02.20 | 47°29`56`` 9°13`51`` | heavy rain (-1-5°C) | 470 | river | Thur | + |
| L_43 | 11.02.20 | 47°24`52`` 9°12`3`` | heavy rain (-1-5°C) | 570 | river | Glatt | + |
| L_44 | 11.02.20 | 47°19`25`` 9°5`6`` | heavy rain (-1-5°C) | 600 | stream | Lederbach | - |
| L_45 | 11.02.20 | 47°13`30`` 9°11`20`` | heavy rain (-1-5°C) | 730 | river | Thur | + |
| L_46 | 11.02.20 | 47°19`44`` 9°17`43`` | heavy rain (-1-5°C) | 800 | stream | Urnäsch | + |
| L_47 | 11.02.20 | 47°20`49`` 9°23`48`` | heavy rain (-1-5°C) | 780 | river | Sitter | + |
| L_48 | 11.02.20 | 47°22`12`` 9°34`11`` | heavy rain (-1-5°C) | 420 | stream | Rietaach | + |
| L_49 | 11.02.20 | 47°29`31`` 9°33`56`` | heavy rain (-1-5°C) | 400 | river | Rhein | + |
| L_50 | 11.02.20 | 47°21`16` 9°7`45`` | heavy rain (-1-5°C) | 630 | river | Necker | + |
| L_51 | 11.02.20 | 47°22`0` 9°24`9`` | heavy rain (-1-5°C) | 800 | stream | Rotbach | + |

| Water sample ID* | Sampling date (dd.mm.yy) | Location of isolation (DMS) | Weather conditions (temperature) | Altitude (m) | Type of water body | Name of water body | Downstream of WWTP** |
|---|---|---|---|---|---|---|---|
| L_52 | 11.02.20 | 47°33`23`` 9°22`42`` | heavy rain (-1-5°C) | 400 | river | Aach | + |
| L_53 | 17.02.20 | 47°19`35`` 7°50`32`` | cloudy (6-12°C) | 420 | river | Dünner | + |
| L_54 | 17.02.20 | 47°18`54`` 7°53`43`` | cloudy (6-12°C) | 400 | river | Wigger | - |
| L_55 | 17.02.20 | 47°15`3`` 7°46`14`` | cloudy (6-12°C) | 430 | river | Aare | + |
| L_56 | 17.02.20 | 47°12`17`` 7°41`52`` | cloudy (6-12°C) | 450 | stream | Önz | + |
| L_57 | 17.02.20 | 47°6`35`` 7°32`8`` | cloudy (6-12°C) | 480 | stream | Aefligen | + |
| L_58 | 17.02.20 | 47°1`28`` 7°38`52`` | cloudy (6-12°C) | 560 | river | Emme | + |
| L_59 | 17.02.20 | 46°56`55`` 7°45`23`` | cloudy (6-12°C) | 650 | stream | Ilfis | + |
| L_60 | 17.02.20 | 46°54`18`` 7°55`51`` | cloudy (6-12°C) | 830 | stream | Lombach | - |
| L_61 | 17.02.20 | 47°1`25`` 8°4`2`` | cloudy (6-12°C) | 610 | river | Kleine Emme | + |
| L_62 | 17.02.20 | 47°5`55`` 7°57`31`` | cloudy (6-12°C) | 620 | stream | Nollentalbach | - |
| L_63 | 17.02.20 | 47°13`57`` 7°58`12`` | rainy (6-12°C) | 460 | stream | Wigger | + |
| L_64 | 16.02.20 | 47° 6`9`` 9°20`6`` | fair (6-12°C) | 435 | stream | Schils | + |
| L_65 | 16.02.20 | 47°6`15`` 9°20`5`` | fair (6-12°C) | 430 | stream | Seez | + |
| L_66 | 16.02.20 | 47°5`52`` 9°18`21`` | fair (6-12°C) | 960 | stream | Tobelbach | - |
| L_67 | 16.02.20 | 47°4`59`` 9°19`3`` | fair (6-12°C) | 950 | stream | Ruslenbach | - |
| L_68 | 16.02.20 | 46°54`59`` 9°45`29`` | fair (6-12°C) | 790 | river | Landquart | + |
| L_69 | 17.02.20 | 47°6`1`` 7°57`45`` | cloudy (6-12°C) | 610 | stream | Wigger | - |
| L_70 | 24.02.20 | 47°18`58`` 8°3`5`` | cloudy (12-15° C) | 450 | river | Suhre | + |
| L_71 | 24.02.20 | 47°13`41`` 8°4`32`` | cloudy (12-15° C) | 480 | river | Suhre | + |
| L_72 | 24.02.20 | 47°17`21`` 8°8`1`` | cloudy (12-15° C) | 490 | stream | Wyna | + |
| L_73 | 24.02.20 | 47°10`43`` 8°16`39`` | cloudy (12-15° C) | 470 | inland canal | Ron | + |
| L_74 | 24.02.20 | 47° 4`15`` 8° 17`32`` | cloudy (12-15° C) | 430 | river | Reuss | + |
| L_75 | 24.02.20 | 46°56`46`` 8°16`0`` | cloudy (12-15° C) | 430 | river | Sarner Aa | + |
| L_76 | 24.02.20 | 46°58`17`` 8°20`26`` | fair (12-18°C) | 440 | inland canal | Vorlauter | + |
| L_77 | 24.02.20 | 46°58`39`` 8°25`3`` | fair (12-18°C) | 440 | stream | Engelburger Aa | + |

| Water sample ID* | Sampling date (dd.mm.yy) | Location of isolation (DMS) | Weather conditions (temperature) | Altitude (m) | Type of water body | Name of water body | Downstream of WWTP** |
|---|---|---|---|---|---|---|---|
| L_78 | 24.02.20 | 46°57`30`` 8°31`33`` | fair (12-18°C) | 720 | stream | Choltalbach | - |
| L_79 | 24.02.20 | 46°53`17`` 8°37`6`` | fair (12-18°C) | 440 | inland canal | Giessenkanal | + |
| L_80 | 24.02.20 | 46°53`0`` 8°37`7`` | fair (12-18°C) | 440 | river | Reuss | - |
| L_81 | 24.02.20 | 47°0`45`` 8°37`10`` | fair (12-18°C) | 440 | stream | Seeweren | + |
| L_82 | 24.02.20 | 47°6`41`` 8°27`4`` | fair (12-18°C) | 430 | stream | Elibach | - |
| L_83 | 24.02.20 | 47°11`58` 8°26`27`` | fair (12-18°C) | 430 | stream | Bach Lorze | - |
| L_84 | 24.02.20 | 47°12`9`` 8°25`57`` | fair (12-18°C) | 390 | inland canal | Kanal Lorze | + |
| L_85 | 24.02.20 | 47°0`42`` 8°37`55`` | fair (12-18°C) | 440 | river | Muota | - |
| L_86 | 02.03.20 | 47°13`14`` 7°34`25`` | cloudy (2-8°C) | 420 | river | Aare | + |
| L_87 | 02.03.20 | 47°10`21`` 7°25`14`` | cloudy (2-8°C) | 430 | river | Aare | + |
| L_88 | 02.03.20 | 47°10`33`` 7°24`59`` | cloudy (2-8°C) | 430 | stream | Witibachkanal | + |
| L_89 | 02.03.20 | 47°7`10`` 7°15`31`` | cloudy (2-8°C) | 410 | inland canal | Büren | + |
| L_90 | 02.03.20 | 47°11`16`` 7°12`17`` | cloudy (2-8°C) | 630 | stream | Schüss | + |
| L_91 | 02.03.20 | 47°13`32`` 7°7`48`` | cloudy (2-8°C) | 860 | stream | Trame | + |
| L_92 | 02.03.20 | 47°9`47`` 7°1`56`` | cloudy (2-8°C) | 730 | stream | Schüss | + |
| L_93 | 02.03.20 | 47°7`35`` 6°51`25`` | cloudy (2-8°C) | 960 | stream | la Ronde | + |
| L_94 | 02.03.20 | 47°1`10`` 6°53`46`` | cloudy (2-8°C) | 730 | stream | la Sagnetanna | + |
| L_95 | 02.03.20 | 47°0`38`` 7°1`55`` | cloudy (2-8°C) | 430 | inland canal | Zihlkana | + |
| L_96 | 02.03.20 | 46°58`0`` 7°10`52`` | cloudy (2-8°C) | 430 | inland canal | Maria Brunnenbach | + |
| L_97 | 02.03.20 | 47°5`32`` 7°18`28`` | cloudy (2-8°C) | 440 | river | Alte Aare | + |
| L_98 | 02.03.20 | 46°57`1`` 7°9`43`` | cloudy (2-8°C) | 440 | inland canal | Galmizkanal | + |
| L_99 | 02.03.20 | 46°42`16`` 8°51`41`` | fair (0-7°C) | 1100 | stream | Acletta | + |
| L_100 | 02.03.20 | 46°46`49`` 9°13`49`` | fair (0-7°C) | 680 | river | Vorderrhein | + |
| L_101 | 02.03.20 | 47°10`35`` 8°54`39`` | rainy (3-7°C) | 440 | stream | Färlibach | - |
| L102 | 09.03.20 | 47°26`17`` 8°33`29`` | rain (4-7°C) | 420 | river | Glatt | + |
| L103 | 09.03.20 | 47°27`2`` 8°25`12`` | rain (4-7°C) | 420 | stream | Furtbach | + |

| Water sample ID* | Sampling date (dd.mm.yy) | Location of isolation (DMS) | Weather conditions (temperature) | Altitude (m) | Type of water body | Name of water body | Downstream of WWTP** |
|---|---|---|---|---|---|---|---|
| L104 | 09.03.20 | 47°24`19`` 8°28`13`` | rain (4-7°C) | 390 | river | Limmat | + |
| L105 | 09.03.20 | 47°21`21`` 8°20`45`` | rain (4-7°C) | 370 | river | Reuss | + |
| L106 | 09.03.20 | 47°21`49`` 8°25`9`` | rain (4-7°C) | 450 | stream | Reppisch | + |
| L107 | 09.03.20 | 47°17`28`` 8°25`59`` | rain (4-7°C) | 460 | stream | Jonen | + |
| L108 | 09.03.20 | 47°12`51`` 8°34`53`` | rain (4-7°C) | 540 | stream | Sarbach | + |
| L109 | 09.03.20 | 47° 8`34`` 8°45`5`` | rain (4-7°C) | 860 | river | Alp | + |
| L110 | 09.03.20 | 47°10`54`` 8°57`42`` | rain (4-7°C) | 410 | inland canal | Wildbachkanal | + |
| L111 | 09.03.20 | 47°10`3`` 9°0`51`` | rain (4-7°C) | 430 | inland canal | Linthkanal | + |
| L112 | 09.03.20 | 47°5`53`` 9°3`49`` | rain (4-7°C) | 430 | stream | Mülibach | - |
| L113 | 09.03.20 | 47°3`31`` 9°3`8`` | rain (4-7°C) | 460 | stream | Löntsch | - |
| L114 | 09.03.20 | 47°2`35`` 9°4`17`` | rain (4-7°C) | 470 | river | Linth | - |
| L115 | 09.03.20 | 46°59`3`` 9°8`41`` | rain (4-7°C) | 770 | river | Senft | + |
| L116 | 09.03.20 | 46°56`14`` 9°0`55`` | rain (4-7°C) | 590 | river | Linth | - |
| L117 | 09.03.20 | 47°14`2`` 8°55`29`` | rain (4-7°C) | 400 | stream | Wagnerbach | + |
| L118 | 08.03.20 | 46°42`41`` 9°32`22`` | rain (4-7°C) | 1600 | stream | Sporz | - |
| L119 | 08.03.20 | 46°42`31`` 9°33`7`` | rain (4-7°C) | 1400 | stream | Rain digl Lai | + |
| L120 | 08.03.20 | 46°45`14`` 9°47`12`` | rain (4-7°C) | 1440 | river | Landwasser | + |
| L121 | 09.03.20 | 47°25`33`` 8°32`30`` | rain (4-7°C) | 430 | stream | Chatzenbach | - |
| L122 | 15.03.20 | 46°30`49`` 9°51`47`` | fair (2-13°C) | 1710 | river | Inn | + |
| L123 | 15.03.20 | 46°30`30`` 9°52`53`` | fair (2-13°C) | 1730 | river | Flaz repp | - |
| L124 | 15.03.20 | 46°57`43`` 10°24`53`` | fair (2-13°C) | 1560 | stream | Schergenbach | - |
| L125 | 15.03.20 | 46°47`57`` 10°19`9`` | fair (2-13°C) | 1170 | river | Inn | + |
| L126 | 15.03.20 | 46°46`1`` 10°6`37`` | fair (2-13°C) | 1420 | stream | Lavinuoz | - |
| L127 | 16.03.20 | 46°57`14`` 6°43`35`` | fair (-1-10°C) | 730 | stream | Noiraigue | + |
| L128 | 16.03.20 | 46°54`60`` 6°36`39`` | fair (-1-10°C) | 730 | river | Areuse | + |
| L129 | 16.03.20 | 46°48`32`` 6°33`1`` | fair (-1-10°C) | 580 | stream | L`Arnon | + |

| Water sample ID* | Sampling date (dd.mm.yy) | Location of isolation (DMS) | Weather conditions (temperature) | Altitude (m) | Type of water body | Name of water body | Downstream of WWTP** |
|---|---|---|---|---|---|---|---|
| L130 | 16.03.20 | 46°47`3`` 6°35`6`` | fair (-1-10°C) | 430 | stream | le Bey | + |
| L131 | 16.03.20 | 46°42`56`` 6°23`21`` | fair (-1-10°C) | 740 | river | l`Orbre | + |
| L132 | 16.03.20 | 46°36`11`` 6°14`25`` | fair (-1-10°C) | 1010 | river | l`Orbre | + |
| L133 | 16.03.20 | 46°24`49`` 6°15`20`` | fair (-1-10°C) | 430 | river | Promenthoux | - |
| L134 | 16.03.20 | 46°16`35`` 6° 9`59`` | fair (-1-10°C) | 380 | stream | la Versoix | - |
| L135 | 16.03.20 | 46°11`40`` 6°5`19`` | fair (-1-10°C) | 350 | river | Rhone | + |
| L136 | 16.03.20 | 46°10`36`` 6°0`28`` | fair (-1-10°C) | 350 | river | Rhone | + |
| L137 | 16.03.20 | 46°10`57`` 6°0`36`` | fair (-1-16°C) | 350 | river | Allondon | - |
| L138 | 16.03.20 | 46°10`59`` 6°11`1`` | fair (-1-16°C) | 400 | stream | Seymaz | - |
| L139 | 16.03.20 | 46°10`45`` 6°10`54`` | fair (-1-16°C) | 390 | river | Arve | + |
| L140 | 16.03.20 | 46°32`36`` 6°33`2`` | fair (-1-16°C) | 390 | river | Venoge | + |
| L141 | 16.03.20 | 46°38`26`` 6°37`29`` | fair (-1-16°C) | 600 | river | le Talent | + |
| L142 | 16.03.20 | 46°50`7`` 6°56`16`` | fair (-1-16°C) | 450 | stream | Broye | + |
| L143 | 16.03.20 | 46°47`3`` 7°6`57`` | fair (-1-16°C) | 580 | river | la Glane | + |
| L144 | 17.03.20 | 46°48`54`` 7°9`52`` | fair (-1-8°C) | 535 | river | Saane | + |
| L145 | 17.03.20 | 46°55`5`` 7°14`23`` | fair (-1-8°C) | 480 | river | Saane | + |
| L146 | 17.03.20 | 46°54`15`` 7°14`9`` | fair (-1-8°C) | 490 | river | Sense | - |
| L147 | 17.03.20 | 46°58`23`` 7°25`40`` | fair (-1-8°C) | 470 | river | Aare | + |
| L148 | 17.03.20 | 46°52`48`` 7°32`45`` | fair (-1-8°C) | 520 | river | Giessen | + |
| L149 | 17.03.20 | 46°46`52`` 7°35`56`` | fair (5-18°C) | 550 | river | Aare | + |
| L150 | 17.03.20 | 46°39`14`` 7°34`32`` | fair (5-18°C) | 670 | stream | Simmen | - |
| L151 | 17.03.20 | 46°35`51`` 7°39`39`` | fair (5-18°C) | 750 | river | Entschligen | + |
| L152 | 17.03.20 | 46°40`59`` 7°39`35`` | fair (5-18°C) | 620 | river | Kander | - |
| L153 | 17.03.20 | 46°39`11`` 7°52`12`` | fair (5-18°C) | 610 | river | Lütschine | - |
| L154 | 17.03.20 | 46°40`28`` 7°50`36`` | fair (5-18°C) | 560 | river | Aare | + |
| L155 | 17.03.20 | 46°43`59`` 8°9`37`` | fair (5-18°C) | 600 | river | Aare | + |

| Water sample ID* | Sampling date (dd.mm.yy) | Location of isolation (DMS) | Weather conditions (temperature) | Altitude (m) | Type of water body | Name of water body | Downstream of WWTP** |
|---|---|---|---|---|---|---|---|
| L156 | 17.03.20 | 46°49`57`` 8° 10`45`` | fair (5-18°C) | 480 | stream | Laui | - |
| L157 | 03.05.20 | 47°13`2`` 9°29`60`` | fair (4-14°C) | 440 | river | Rhein | + |
| L158 | 03.05.20 | 47°1`24`` 9°30`0`` | fair (4-14°C) | 500 | river | Rhein | + |
| L159 | 03.05.20 | 46°52`18`` 9°31`42`` | fair (4-14°C) | 510 | river | Rhein | + |
| L160 | 03.05.20 | 46°33`18`` 9°19`55`` | fair (1-12°C) | 1440 | river | Hinterrhein | + |
| L161 | 03.05.20 | 46°17`35`` 9°10`48`` | fair (6-20°C) | 400 | river | Moesa | + |
| L162 | 03.05.20 | 46°10`30`` 8°59`28`` | fair (6-20°C) | 220 | river | Ticino | + |
| L163 | 03.05.20 | 46°2`8`` 8°58`20`` | fair (6-20°C) | 320 | river | Cassarate | + |
| L164 | 03.05.20 | 46°0`23`` 8°54`48`` | fair (6-20°C) | 280 | river | Vedeggio | + |
| L165 | 03.05.20 | 45°53`33`` 8°58`24`` | fair (6-20°C) | 280 | river | Laveggio | + |
| L166 | 03.05.20 | 45°50`31`` 9°2`13`` | fair (6-24°C) | 230 | river | Breggia | + |
| L167 | 03.05.20 | 46°10`15`` 8°51`31`` | fair (6-24°C) | 200 | river | Versasca | - |
| L168 | 03.05.20 | 46°10`47`` 8° 45`3`` | fair (6-24°C) | 220 | river | Melezza | - |
| L169 | 03.05.20 | 46°16`48`` 8°39`59`` | fair (6-24°C) | 360 | river | Maggia | - |
| L170 | 03.05.20 | 46°19`52`` 8°58`29`` | fair (6-24°C) | 270 | river | Ticino | + |
| L171 | 03.05.20 | 46°29`21`` 8°44`39`` | fair (6-20°C) | 940 | river | Ticino | + |
| L172 | 03.05.20 | 46°31`29`` 8°37`36`` | Fair(6-18°C) | 1100 | river | Canaria | + |
| L173 | 03.05.20 | 46°46`19`` 8°40`11`` | fair (5-15°C) | 510 | river | Kärstelenbach | - |
| L174 | 12.05.20 | 46°38`14`` 7°3`20`` | light rain (3-6°C) | 730 | stream | La Sionge | - |
| L175 | 12.05.20 | 46°36`40`` 7°5`29`` | light rain (3-6°C) | 680 | river | Saane | + |
| L176 | 12.05.20 | 46°28`8`` 6°50`53`` | light rain (3-6°C) | 400 | river | la Veveyse | - |
| L177 | 12.05.20 | 46°22`30`` 6°55`45`` | light rain (3-6°C) | 380 | stream | Eau Froide | + |
| L178 | 12.05.20 | 46°13`2 7°0`29`` | light rain (3-6°C) | 400 | river | Rhone | + |
| L179 | 12.05.20 | 46°7`11` 7°4`10`` | light rain (3-6°C) | 470 | inland canal | Canal du Syndicat | + |
| L180 | 12.05.20 | 46°7`7`` 7°4`5`` | light rain (3-6°C) | 460 | river | la Drance | - |
| L181 | 12.05.20 | 46°13`12`` 7°21`36`` | light rain (3-6°C) | 480 | river | Rhone | + |

| Water sample ID* | Sampling date (dd.mm.yy) | Location of isolation (DMS) | Weather conditions (temperature) | Altitude (m) | Type of water body | Name of water body | Downstream of WWTP** |
|---|---|---|---|---|---|---|---|
| L182 | 12.05.20 | 46°10`53`` 7°25`4`` | light rain (3-6°C) | 500 | river | la Borgne | + |
| L183 | 12.05.20 | 46°16`35`` 7°30`30`` | light rain (3-6°C) | 520 | river | Rhone | + |
| L184 | 12.05.20 | 46°18`23`` 7°41`33`` | light rain (3-6°C) | 620 | stream | Turtmänna | - |
| L185 | 12.05.20 | 46°18`17`` 7°51`21`` | cloudy (5-13°C) | 640 | river | Rhone | + |
| L186 | 12.05.20 | 46°14`43`` 7°52`30`` | cloudy (5-13°C) | 650 | river | Vispa | + |
| L187 | 12.05.20 | 46°18`25`` 7°56`50`` | cloudy (5-13°C) | 660 | river | Rhone | + |
| L188 | 12.05.20 | 46°23`30`` 8°7`32`` | cloudy (5-13°C) | 990 | river | Rhone | + |
| L189 | 12.05.20 | 46°29`11`` 8°15`46`` | cloudy (5-13°C) | 1380 | stream | Mistigerbach | - |
| L190 | 12.05.20 | 46°32`10`` 8°21`26`` | cloudy (5-13°C) | 1360 | stream | Goneri | - |
| L191 | 12.05.20 | 46°38`32`` 8°35`26`` | cloudy (5-13°C) | 1430 | river | Reuss | + |

* Cells highlighted in grey indicate samples that tested positive for *L. monocytogenes*. Strain ID and clonal complex (CC) are indicated in brackets.

** WWTP, wastewater treatment plant; +, WWTP located upstream; -, no WWTP in the vicinity.

**Table S2.** Internalin A amino acid sequence comparison among 25 *Listeria monocytogenes* isolated from surface water

| Amino acid position and substitution[1] | Type of amino acid substitution | Strain ID | Remarks |
|---|---|---|---|
| S32N | Non-conservative | L41 | |
| V44I | Conservative | L58, L105,L124, L49 | CC4 only |
| A51T | Non-conservative | L86, L72, L50, L137, L180, L51, L138, L36 | |
| L94V | Conservative | L28, L44, L42, L128-3, L174, L49, L58, L105, L124, L12, L164, L52, L41, L72, L50 | |
| D118N | Non-conservative | L28, L44, L42, L128-3, L174, L49, L58, L105, L124, L12, L164, L52, L41, L72, L50 | |
| S142T | Conservative | L42, L44, L128-3, L28, L174 | CC1 only |
| L157I | Conservative | L86, L137, L180, L51, L138 | |
| S187N | Non-conservative | L52, L58, L105,L124, L49, L72, L50 | |
| A454T | Non-conservative | L28, L44, L42, L128-3, L174, L49, L58, L105, L124, L12, L164, L52, L41,L50, L72, L36, L137, L180, L51, L138, | |
| N474S | Non-conservative | L28, L44, L42, L128-3, L174, L49, L58, L105, L124, L12, L164, L52, L41, L36, L137, L180, L51, L138, | |
| S476P | Non-conservative | L28, L44, L42, L128-3, L174, L49, L58, L105, L124, L12, L164, L52, L41, L36, L137, L180, L51, L138, | |
| A500V | Conservative | L28, L44, L42, L128-3, L174, L49, L58, L105, L124, L12, L164, L52, L41,L50, L72, L36, L137, L180, L51, L138, L57, L111, L127, L188 | |
| Y530H | Non-conservative | L28, L44, L42, L128-3, L174, L49, L58, L105, L124, L12, L164, L52, L41, L36, L137, L180, L51, L138, | |
| I533V | Conservative | L12, L164, L137, L180, L138, L36, L51 | |
| K539Q | Non-conservative | L137, L180, L138, L51 | |
| N544K | Non-conservative | L12, L164 | CC6 only |
| D558N | Non-conservative | L137, L180, L138, L51, L36, L28, L44, L42, L128-3, L174, L49, L58, L105, L124, L12, L164, L52 | |

| Amino acid position and substitution[1] | Type of amino acid substitution | Strain ID | Remarks |
|---|---|---|---|
| L572F | Non-conservative | L12, L164, L137, L180, L138, L36, L51 | |
| E573D | Conservative | L127, L188, L111, L57, L86, L72, L50 | |
| P594A | Non-conservative | L127, L188, L111, L57, L86, L72, L50 | |
| I644V | Conservative | L127, L188, L111, L57, L86 | |
| T648S | Conservative | L36, L137, L180, L51, L138, L86, L57, L111, L127, L188, L28, L44, L42, L128-3, L174, L49, L58, L105, L124, L12, L164, L52, L41 | |
| T652A | Non-conservative | L127, L188, L111, L57, L86 | |
| T664A | Non-conservative | L28, L44, L42, L128-3, L174, L49, L58, L105, L124, L12, L164, L52, L41, L137, L180, L36 | |
| A671T | Non-conservative | L12, L164 | CC6 only |
| -741N | Non-conservative | L12, L164 | CC6 only |
| -742T | Non-conservative | L12, L164 | CC6 only |
| -743S | Non-conservative | L12, L164 | CC6 only |
| D764E | Conservative | L86, L51, L138 | |
| Y774D | Non-conservative | L58, L105,L124, L49, L12, L164 | CC4 and CC6 |

[1] Amino acid positions are relative to *L. monocytogenes* EGDe InlA protein.

**Table S3:** Presence or absence of virulence factor genes among 25 *Listeria monocytogenes* isolated from surface water

| | | | Strain lineage, clonal complex, serotype and ID | | | | | | | | | | | | | | | | | | | | | | | | | | | | | |
|---|---|---|---|---|---|---|---|---|---|---|---|---|---|---|---|---|---|---|---|---|---|---|---|---|---|---|---|---|---|---|---|---|
| | | | Lineage I | | | | | | | | | | | | | | | Lineage II | | | | | | | | | | | | | | |
| | | | CC1, 4b | CC4, 4b | CC1, 4b | CC1, 4b | CC1, 4b | CC1, 4b | CC1, 4b | CC4, 4b | CC4, 4b | CC4, 4b | CC4, 4b | CC6, 4b | CC6, 4b | 1/2b | CC59, 1/2b | CC9, 1/2a | CC8, 1/2a | CC7, 1/2a | CC7, 1/2a | CC11, 1/2 | CC29, 1/2 | CC29, 1/2 | CC37, 1/2 | CC37, 1/2 | CC14, ST9 | CC412, 1/ | CC412, 1/ | CC412, 1/ | CC412, 1/ | CC412, 1/2a |
| **Locus** | **Allele** | **Length** | **LL195** | **N2306** | **L28** | **L44** | **L42** | **L128-3** | **L174** | **L49** | **L58** | **L105** | **L124** | **L12** | **L164** | **L52** | **L41** | **EGDe** | **N1546** | **L50** | **L72** | **L36** | **L137** | **L180** | **L51** | **L138** | **L86** | **N12-1273** | **L57** | **L111** | **L127** | **L188** |
| prfA (lmo0200) | 4 | 714 | 1 | 1 | 1 | 1 | 1 | 1 | 1 | 1 | 1 | 1 | 1 | 1 | 1 | 1 | 1 | 1 | 1 | 1 | 1 | 1 | 1 | 1 | 1 | 1 | 1 | 1 | 1 | 1 | 1 | 1 |
| plcA (lmo0201) | 9 | 954 | 1 | 1 | 1 | 1 | 1 | 1 | 1 | 1 | 1 | 1 | 1 | 1 | 1 | 1 | 1 | 1 | 1 | 1 | 1 | 1 | 1 | 1 | 1 | 1 | 1 | 1 | 1 | 1 | 1 | 1 |
| hly (lmo0202) | 5 | 1590 | 1 | 1 | 1 | 1 | 1 | 1 | 1 | 1 | 1 | 1 | 1 | 1 | 1 | 1 | 1 | 1 | 1 | 1 | 1 | 1 | 1 | 1 | 1 | 1 | 1 | 1 | 1 | 1 | 1 | 1 |
| mpl (lmo0203) | 6 | 1533 | 1 | 1 | 1 | 1 | 1 | 1 | 1 | 1 | 1 | 1 | 1 | 1 | 1 | 1 | 1 | 1 | 1 | 1 | 1 | 1 | 1 | 1 | 1 | 1 | 1 | 1 | 1 | 1 | 1 | 1 |
| actA (lmo0204) | 119 | 1815 | 1 | 1 | 1 | 1 | 1 | 1 | 1 | 1 | 1 | 1 | 1 | 1 | 1 | 1 | 1 | 1 | 1 | 1 | 1 | 1 | 1 | 1 | 1 | 1 | 1 | 1 | 1 | 1 | 1 | 1 |
| plcB (lmo0205) | 4 | 870 | 1 | 1 | 1 | 1 | 1 | 1 | 1 | 1 | 1 | 1 | 1 | 1 | 1 | 1 | 1 | 1 | 1 | 1 | 1 | 1 | 1 | 1 | 1 | 1 | 1 | 1 | 1 | 1 | 1 | 1 |
| LIPI3_llsA (LMOf2365_1112a) | 1 | 150 | 1 | 1 | 1 | 1 | 1 | 1 | 1 | 1 | 1 | 1 | 1 | 1 | 1 | 1 | 0 | 0 | 0 | 0 | 0 | 0 | 0 | 0 | 0 | 0 | 0 | 0 | 0 | 0 | 0 | 0 |
| LIPI3_llsG (LMOf2365_1113) | 1 | 888 | 1 | 1 | 1 | 1 | 1 | 1 | 1 | 1 | 1 | 1 | 1 | 1 | 1 | 1 | 0 | 0 | 0 | 0 | 0 | 0 | 0 | 0 | 0 | 0 | 0 | 0 | 0 | 0 | 0 | 0 |
| LIPI3_llsH (LMOf2365_1114) | 2 | 750 | 1 | 1 | 1 | 1 | 1 | 1 | 1 | 1 | 1 | 1 | 1 | 1 | 1 | 1 | 0 | 0 | 0 | 0 | 0 | 0 | 0 | 0 | 0 | 0 | 0 | 0 | 0 | 0 | 0 | 0 |
| LIPI3_llsX (LMOf2365_1115) | 2 | 318 | 1 | 1 | 1 | 1 | 1 | 1 | 1 | 1 | 1 | 1 | 1 | 1 | 1 | 1 | 0 | 0 | 0 | 0 | 0 | 0 | 0 | 0 | 0 | 0 | 0 | 0 | 0 | 0 | 0 | 0 |
| LIPI3_llsB (LMOf2365_1116) | 3 | 876 | 1 | 1 | 1 | 1 | 1 | 1 | 1 | 1 | 1 | 1 | 1 | 1 | 1 | 1 | 0 | 0 | 0 | 0 | 0 | 0 | 0 | 0 | 0 | 0 | 0 | 0 | 0 | 0 | 0 | 0 |
| LIPI3_llsY (LMOf2365_1117) | 3 | 918 | 1 | 1 | 1 | 1 | 1 | 1 | 1 | 1 | 1 | 1 | 1 | 1 | 1 | 1 | 0 | 0 | 0 | 0 | 0 | 0 | 0 | 0 | 0 | 0 | 0 | 0 | 0 | 0 | 0 | 0 |
| LIPI3_llsD (LMOf2365_1118) | 3 | 1314 | 1 | 1 | 1 | 1 | 1 | 1 | 1 | 1 | 1 | 1 | 1 | 1 | 1 | 1 | 0 | 0 | 0 | 0 | 0 | 0 | 0 | 0 | 0 | 0 | 0 | 0 | 0 | 0 | 0 | 0 |
| LIPI3_llsP (LMOf2365_1119) | 2 | 588 | 1 | 1 | 1 | 1 | 1 | 1 | 1 | 1 | 1 | 1 | 1 | 1 | 1 | 1 | 0 | 0 | 0 | 0 | 0 | 0 | 0 | 0 | 0 | 0 | 0 | 0 | 0 | 0 | 0 | 0 |
| inlA (lmo0433) | 7 | 2403 | 1 | 1 | 1 | 1 | 1 | 1 | 1 | 1 | 1 | 1 | 1 | 1 | 1 | 1 | 1 | 1 | 1 | 1 | 1 | 1 | 1 | 1 | 1 | 1 | 1 | 1 | 1 | 1 | 1 | 1 |
| inlB (lmo0434) | 5 | 1893 | 1 | 1 | 1 | 1 | 1 | 1 | 1 | 1 | 1 | 1 | 1 | 1 | 1 | 1 | 1 | 1 | 1 | 1 | 1 | 1 | 1 | 1 | 1 | 1 | 1 | 1 | 1 | 1 | 1 | 1 |
| inlC (lmo1786) | 5 | 891 | 1 | 1 | 1 | 1 | 1 | 1 | 1 | 1 | 1 | 1 | 1 | 1 | 1 | 1 | 1 | 1 | 1 | 1 | 1 | 1 | 1 | 1 | 1 | 1 | 1 | 1 | 1 | 1 | 1 | 1 |
| inlE (lmo0264) | 4 | 1500 | 1 | 1 | 1 | 1 | 1 | 1 | 1 | 1 | 1 | 1 | 1 | 1 | 1 | 1 | 1 | 1 | 1 | 1 | 1 | 1 | 1 | 1 | 1 | 1 | 1 | 1 | 1 | 1 | 1 | 1 |
| inlF (lmo0409) | 12 | 2472 | 1 | 1 | 1 | 1 | 1 | 1 | 1 | 1 | 1 | 1 | 1 | 1 | 1 | 1 | 1 | 1 | 1 | 1 | 1 | 1 | 1 | 1 | 1 | 1 | 1 | 1 | 1 | 1 | 1 | 1 |
| inlG (lmo0262) | 3 | 1473 | 0 | 0 | 0 | 0 | 0 | 0 | 0 | 0 | 0 | 0 | 0 | 1 | 1 | 0 | 0 | 1 | 1 | 1 | 1 | 1 | 1 | 1 | 1 | 1 | 1 | 1 | 1 | 1 | 1 | 1 |
| inlH (lmo0263) | 4 | 1647 | 1 | 1 | 1 | 1 | 1 | 1 | 1 | 1 | 1 | 1 | 1 | 1 | 1 | 1 | 1 | 1 | 1 | 1 | 1 | 1 | 1 | 1 | 1 | 1 | 1 | 1 | 1 | 1 | 1 | 1 |
| inlJ (lmo2821) | 4 | 2541 | 1 | 1 | 1 | 1 | 1 | 1 | 1 | 1 | 1 | 1 | 1 | 1 | 1 | 1 | 1 | 1 | 1 | 1 | 1 | 1 | 1 | 1 | 1 | 1 | 1 | 1 | 1 | 1 | 1 | 1 |
| inlK (lmo1290) | 4 | 1821 | 1 | 1 | 1 | 1 | 1 | 1 | 1 | 1 | 1 | 1 | 1 | 1 | 1 | 1 | 1 | 1 | 1 | 1 | 1 | 1 | 1 | 1 | 1 | 1 | 1 | 1 | 1 | 1 | 1 | 1 |
| agrA (lmo0051) | 3 | 729 | 1 | 1 | 1 | 1 | 1 | 1 | 1 | 1 | 1 | 1 | 1 | 1 | 1 | 1 | 1 | 1 | 1 | 1 | 1 | 1 | 1 | 0 | 1 | 1 | 1 | 1 | 1 | 1 | 1 | 1 |
| agrC (lmo0050) | 4 | 1296 | 1 | 1 | 1 | 1 | 1 | 1 | 1 | 1 | 1 | 1 | 1 | 1 | 1 | 1 | 1 | 2 | 1 | 2 | 2 | 1 | 1 | 1 | 1 | 1 | 2 | 1 | 1 | 1 | 1 | 1 |
| ami (lmo2558) | 48 | 2754 | 0 | 0 | 0 | 0 | 0 | 0 | 0 | 0 | 0 | 0 | 0 | 0 | 0 | 0 | 1 | 1 | 1 | 1 | 1 | 1 | 1 | 1 | 1 | 1 | 1 | 1 | 1 | 1 | 1 | 1 |
| aut (lmo1076) | 4 | 1719 | 0 | 0 | 0 | 0 | 0 | 0 | 0 | 0 | 0 | 0 | 0 | 0 | 0 | 1 | 1 | 1 | 1 | 1 | 1 | 1 | 1 | 1 | 1 | 1 | 1 | 1 | 0 | 1 | 1 | 1 |
| bsh (lmo2067) | 4 | 978 | 1 | 1 | 1 | 1 | 1 | 1 | 1 | 1 | 1 | 1 | 1 | 1 | 1 | 1 | 1 | 1 | 1 | 1 | 1 | 1 | 1 | 1 | 1 | 1 | 1 | 1 | 1 | 1 | 1 | 1 |
| cheA (lmo0692) | 4 | 1857 | 1 | 1 | 1 | 1 | 1 | 1 | 1 | 1 | 1 | 1 | 1 | 1 | 1 | 1 | 1 | 1 | 1 | 1 | 1 | 1 | 1 | 1 | 1 | 1 | 1 | 1 | 1 | 1 | 1 | 1 |
| cheY (lmo0691) | 3 | 360 | 1 | 1 | 1 | 1 | 1 | 1 | 1 | 1 | 1 | 1 | 1 | 1 | 1 | 1 | 1 | 1 | 1 | 1 | 1 | 1 | 1 | 1 | 1 | 1 | 1 | 1 | 1 | 1 | 1 | 1 |
| cwhA (lmo0582; iap) | 5 | 1428 | 1 | 1 | 1 | 1 | 1 | 1 | 1 | 1 | 1 | 1 | 1 | 1 | 1 | 1 | 1 | 1 | 1 | 1 | 1 | 1 | 1 | 1 | 1 | 1 | 1 | 1 | 1 | 1 | 1 | 1 |
| dltA (lmo0974) | 5 | 1533 | 1 | 1 | 1 | 1 | 1 | 1 | 1 | 1 | 1 | 1 | 1 | 1 | 1 | 1 | 1 | 1 | 1 | 1 | 1 | 1 | 1 | 1 | 1 | 1 | 1 | 1 | 1 | 1 | 1 | 1 |
| fbpA (lmo1829) | 5 | 1713 | 1 | 1 | 1 | 1 | 1 | 1 | 1 | 1 | 1 | 1 | 1 | 1 | 1 | 1 | 1 | 1 | 1 | 1 | 1 | 1 | 1 | 1 | 1 | 1 | 1 | 1 | 1 | 1 | 1 | 1 |
| fur (lmo1956) | 4 | 453 | 1 | 1 | 1 | 1 | 1 | 1 | 1 | 1 | 1 | 1 | 1 | 1 | 1 | 1 | 1 | 1 | 1 | 1 | 1 | 1 | 1 | 1 | 1 | 1 | 1 | 1 | 1 | 1 | 1 | 1 |
| gtcA (lmo2549) | 3 | 438 | 1 | 1 | 1 | 1 | 1 | 1 | 1 | 1 | 1 | 1 | 1 | 1 | 1 | 1 | 1 | 1 | 1 | 1 | 1 | 1 | 1 | 1 | 1 | 1 | 1 | 1 | 1 | 1 | 1 | 1 |
| hpt (lmo0838) | 5 | 1386 | 1 | 1 | 1 | 1 | 1 | 1 | 1 | 1 | 1 | 1 | 1 | 1 | 1 | 1 | 1 | 1 | 1 | 1 | 1 | 1 | 1 | 1 | 1 | 1 | 1 | 1 | 1 | 1 | 1 | 1 |
| lap (lmo1634) | 5 | 2601 | 1 | 1 | 1 | 1 | 1 | 1 | 1 | 1 | 1 | 1 | 1 | 1 | 1 | 1 | 1 | 1 | 1 | 1 | 1 | 1 | 1 | 1 | 1 | 1 | 1 | 1 | 1 | 1 | 1 | 1 |
| lapB (lmo1666) | 348 | 5157 | 1 | 1 | 1 | 1 | 1 | 1 | 1 | 1 | 1 | 1 | 1 | 1 | 1 | 1 | 1 | 1 | 1 | 1 | 1 | 1 | 1 | 1 | 1 | 1 | 1 | 0 | 0 | 0 | 0 | 0 |
| lgt (lmo2482) | 6 | 834 | 1 | 1 | 1 | 1 | 1 | 1 | 1 | 1 | 1 | 1 | 1 | 1 | 1 | 1 | 1 | 1 | 1 | 1 | 1 | 1 | 1 | 1 | 1 | 1 | 1 | 1 | 1 | 1 | 1 | 1 |
| lisK (lmo1378) | 5 | 1452 | 1 | 1 | 1 | 1 | 1 | 1 | 1 | 1 | 1 | 1 | 1 | 1 | 1 | 1 | 1 | 1 | 1 | 1 | 1 | 1 | 1 | 1 | 1 | 1 | 1 | 1 | 1 | 1 | 1 | 1 |
| lisR (lmo1377) | 3 | 681 | 1 | 1 | 1 | 1 | 1 | 1 | 1 | 1 | 1 | 1 | 1 | 1 | 1 | 1 | 1 | 1 | 1 | 1 | 1 | 1 | 1 | 1 | 1 | 1 | 1 | 1 | 1 | 1 | 1 | 1 |
| lntA (lmo0438) | 4 | 618 | 1 | 1 | 1 | 1 | 1 | 1 | 1 | 1 | 1 | 1 | 1 | 1 | 1 | 1 | 1 | 1 | 1 | 1 | 1 | 1 | 1 | 1 | 1 | 1 | 1 | 1 | 1 | 1 | 1 | 1 |
| lpeA (lmo1847) | 4 | 933 | 1 | 1 | 1 | 1 | 1 | 1 | 1 | 1 | 1 | 1 | 1 | 1 | 1 | 1 | 1 | 1 | 1 | 1 | 1 | 1 | 1 | 1 | 1 | 1 | 1 | 1 | 1 | 1 | 1 | 1 |
| lplA1 (lmo0931) | 6 | 996 | 1 | 1 | 1 | 1 | 1 | 1 | 1 | 1 | 1 | 1 | 1 | 1 | 1 | 1 | 1 | 1 | 1 | 1 | 1 | 1 | 1 | 1 | 1 | 1 | 1 | 1 | 1 | 1 | 1 | 1 |
| lspA (lmo1844) | 7 | 465 | 1 | 1 | 1 | 1 | 1 | 1 | 1 | 1 | 1 | 1 | 1 | 1 | 1 | 1 | 1 | 1 | 1 | 1 | 1 | 1 | 1 | 1 | 1 | 1 | 1 | 1 | 1 | 1 | 1 | 1 |
| oatA (lmo1291) | 4 | 1887 | 1 | 1 | 1 | 1 | 1 | 1 | 1 | 1 | 1 | 1 | 1 | 1 | 1 | 1 | 1 | 1 | 1 | 1 | 1 | 1 | 1 | 1 | 1 | 1 | 1 | 1 | 1 | 1 | 1 | 1 |
| oppA (lmo2196) | 5 | 1677 | 1 | 1 | 1 | 1 | 1 | 1 | 1 | 1 | 1 | 1 | 1 | 1 | 1 | 1 | 1 | 1 | 1 | 1 | 1 | 1 | 1 | 1 | 1 | 1 | 1 | 1 | 1 | 1 | 1 | 1 |
| pdgA (lmo0415) | 5 | 1401 | 1 | 1 | 1 | 1 | 1 | 1 | 1 | 1 | 1 | 1 | 1 | 1 | 1 | 1 | 1 | 1 | 1 | 1 | 1 | 1 | 1 | 1 | 1 | 1 | 1 | 1 | 1 | 1 | 1 | 1 |
| prsA2 (lmo2219) | 5 | 882 | 1 | 1 | 1 | 1 | 1 | 1 | 1 | 1 | 1 | 1 | 1 | 1 | 1 | 1 | 1 | 1 | 1 | 1 | 1 | 1 | 1 | 1 | 1 | 1 | 1 | 1 | 1 | 1 | 1 | 1 |
| purQ (lmo1769) | 7 | 2220 | 1 | 1 | 1 | 1 | 1 | 1 | 1 | 1 | 1 | 1 | 1 | 1 | 1 | 1 | 1 | 1 | 1 | 1 | 1 | 1 | 1 | 1 | 1 | 1 | 1 | 1 | 1 | 1 | 1 | 1 |
| srtA (lmo0929) | 4 | 669 | 1 | 1 | 1 | 1 | 1 | 1 | 1 | 1 | 1 | 1 | 1 | 1 | 1 | 1 | 1 | 1 | 1 | 1 | 1 | 1 | 1 | 1 | 1 | 1 | 1 | 1 | 1 | 1 | 1 | 1 |
| srtB (lmo2181) | 11 | 741 | 1 | 1 | 1 | 1 | 1 | 1 | 1 | 1 | 1 | 1 | 1 | 1 | 1 | 1 | 1 | 1 | 1 | 1 | 1 | 1 | 1 | 1 | 1 | 1 | 1 | 1 | 1 | 1 | 1 | 1 |
| stp (lmo1821) | 5 | 759 | 1 | 1 | 1 | 1 | 1 | 1 | 1 | 1 | 1 | 1 | 1 | 1 | 1 | 1 | 1 | 1 | 1 | 1 | 1 | 1 | 1 | 1 | 1 | 1 | 1 | 1 | 1 | 1 | 1 | 1 |
| svpA (lmo2185) | 5 | 1710 | 1 | 1 | 1 | 1 | 1 | 1 | 1 | 1 | 1 | 1 | 1 | 1 | 1 | 1 | 1 | 1 | 1 | 1 | 1 | 1 | 1 | 1 | 1 | 1 | 1 | 1 | 1 | 1 | 1 | 1 |
| tagB (lmo1088) | 6 | 1161 | 0 | 0 | 0 | 0 | 0 | 0 | 0 | 0 | 0 | 0 | 0 | 0 | 0 | 1 | 1 | 1 | 1 | 1 | 1 | 1 | 1 | 1 | 1 | 1 | 1 | 1 | 1 | 1 | 1 | 1 |
| vip (lmo0320) | 52 | 1245 | 1 | 1 | 1 | 1 | 1 | 1 | 0 | 1 | 1 | 1 | 1 | 1 | 1 | 1 | 1 | 1 | 0 | 0 | 0 | 1 | 0 | 0 | 0 | 0 | 1 | 0 | 0 | 0 | 0 | 0 |
| virR (lmo1745) | 4 | 678 | 1 | 1 | 1 | 1 | 1 | 1 | 1 | 1 | 1 | 1 | 1 | 1 | 1 | 1 | 1 | 1 | 1 | 1 | 1 | 1 | 1 | 1 | 1 | 1 | 1 | 1 | 1 | 1 | 1 | 1 |
| virS (lmo1741) | 6 | 1041 | 1 | 1 | 1 | 1 | 1 | 1 | 1 | 1 | 1 | 1 | 1 | 1 | 1 | 1 | 1 | 1 | 1 | 1 | 1 | 1 | 1 | 1 | 1 | 1 | 1 | 1 | 1 | 1 | 1 | 1 |
| aut_IVb (LMOF2365_RS00075) | 1 | 357 | 1 | 1 | 1 | 1 | 1 | 1 | 1 | 1 | 1 | 1 | 1 | 1 | 1 | 0 | 0 | 0 | 0 | 0 | 0 | 0 | 0 | 0 | 0 | 0 | 0 | 0 | 0 | 0 | 0 | 0 |
| comK (LMOf2365_2303) | 3 | 609 | 1 | 1 | 0 | 1 | 1 | 1 | 1 | 1 | 1 | 1 | 1 | 1 | 1 | 1 | 1 | 0 | 0 | 1 | 0 | 1 | 1 | 1 | 1 | 1 | 0 | 1 | 1 | 1 | 1 | 1 |
| gltA (LMOf2365_2740) | 1 | 1854 | 1 | 1 | 1 | 1 | 1 | 1 | 1 | 1 | 1 | 1 | 1 | 1 | 1 | 0 | 0 | 0 | 0 | 0 | 0 | 0 | 0 | 0 | 0 | 0 | 0 | 0 | 0 | 0 | 0 | 0 |
| gltB (LMOf2365_2741) | 1 | 948 | 1 | 1 | 1 | 1 | 1 | 1 | 1 | 1 | 1 | 1 | 1 | 1 | 1 | 0 | 0 | 0 | 0 | 0 | 0 | 0 | 0 | 0 | 0 | 0 | 0 | 0 | 0 | 0 | 0 | 0 |
| inlC2 (LMON_RS01340) | 2 | 1647 | 1 | 1 | 1 | 1 | 1 | 1 | 1 | 1 | 1 | 1 | 1 | 1 | 1 | 1 | 1 | 1 | 1 | 1 | 1 | 1 | 1 | 1 | 1 | 1 | 1 | 1 | 1 | 1 | 1 | 1 |
| inlD (LMON_RS01345) | 5 | 1707 | 1 | 1 | 1 | 1 | 1 | 1 | 1 | 1 | 1 | 1 | 1 | 1 | 1 | 1 | 1 | 0 | 1 | 1 | 1 | 1 | 1 | 1 | 1 | 1 | 1 | 1 | 1 | 1 | 1 | 1 |
| inlL (LMON_RS10535) | 1 | 1881 | 0 | 0 | 0 | 0 | 0 | 0 | 0 | 0 | 0 | 0 | 0 | 0 | 0 | 0 | 0 | 1 | 0 | 1 | 1 | 0 | 1 | 0 | 1 | 1 | 0 | 1 | 1 | 1 | 1 | 1 |
| LM9005581_70009 (LIPI-4) | 1 | 1302 | 0 | 1 | 0 | 0 | 0 | 0 | 0 | 1 | 1 | 1 | 1 | 0 | 0 | 0 | 0 | 0 | 0 | 0 | 0 | 0 | 0 | 0 | 0 | 0 | 0 | 0 | 0 | 0 | 0 | 0 |
| LM9005581_70010 (LIPI-4) | 1 | 357 | 0 | 1 | 0 | 0 | 0 | 0 | 0 | 1 | 1 | 1 | 1 | 0 | 0 | 0 | 0 | 0 | 0 | 0 | 0 | 0 | 0 | 0 | 0 | 0 | 0 | 0 | 0 | 0 | 0 | 0 |
| LM9005581_70011 (LIPI-4) | 1 | 315 | 0 | 1 | 0 | 0 | 0 | 0 | 0 | 1 | 1 | 1 | 1 | 0 | 0 | 0 | 0 | 0 | 0 | 0 | 0 | 0 | 0 | 0 | 0 | 0 | 0 | 0 | 0 | 0 | 0 | 0 |
| LM9005581_70012 (LIPI-4) | 1 | 747 | 0 | 1 | 0 | 0 | 0 | 0 | 0 | 1 | 1 | 1 | 1 | 0 | 0 | 0 | 0 | 0 | 0 | 0 | 0 | 0 | 0 | 0 | 0 | 0 | 0 | 0 | 0 | 0 | 0 | 0 |
| LM9005581_70013 (LIPI-4) | 1 | 1917 | 0 | 1 | 0 | 0 | 0 | 0 | 0 | 1 | 1 | 1 | 1 | 0 | 0 | 0 | 0 | 0 | 0 | 0 | 0 | 0 | 0 | 0 | 0 | 0 | 0 | 0 | 0 | 0 | 0 | 0 |
| LM9005581_70014 (LIPI-4) | 1 | 1308 | 0 | 1 | 0 | 0 | 0 | 0 | 0 | 1 | 1 | 1 | 1 | 0 | 0 | 0 | 0 | 0 | 0 | 0 | 0 | 0 | 0 | 0 | 0 | 0 | 0 | 0 | 0 | 0 | 0 | 0 |
| lmo0333 (inlI) | 18 | 5328 | 1 | 1 | 1 | 1 | 1 | 1 | 1 | 1 | 1 | 1 | 1 | 1 | 1 | 1 | 1 | 1 | 1 | 1 | 1 | 1 | 1 | 1 | 1 | 1 | 1 | 1 | 1 | 1 | 1 | 1 |
| lmo1280 (codY) | 3 | 780 | 1 | 1 | 1 | 1 | 1 | 1 | 1 | 1 | 1 | 1 | 1 | 1 | 1 | 1 | 1 | 1 | 1 | 1 | 1 | 1 | 1 | 1 | 1 | 1 | 1 | 1 | 1 | 1 | 1 | 1 |
| lmo2470 (inlP) | 3 | 1167 | 1 | 1 | 1 | 1 | 1 | 1 | 1 | 1 | 1 | 1 | 1 | 1 | 1 | 1 | 1 | 1 | 1 | 1 | 1 | 1 | 1 | 1 | 1 | 1 | 1 | 1 | 1 | 1 | 1 | 1 |
| lmo2491 (pdeE) | 3 | 648 | 1 | 1 | 1 | 1 | 1 | 1 | 1 | 1 | 1 | 1 | 1 | 1 | 1 | 1 | 1 | 1 | 1 | 1 | 1 | 1 | 1 | 1 | 1 | 1 | 1 | 1 | 1 | 1 | 1 | 1 |
| mdrM (lmo1617) | 2 | 1470 | 1 | 1 | 1 | 1 | 1 | 1 | 1 | 1 | 1 | 1 | 1 | 1 | 1 | 1 | 1 | 1 | 1 | 1 | 1 | 1 | 1 | 1 | 1 | 1 | 1 | 1 | 1 | 1 | 1 | 1 |
| **Total number of genes** | | | **65** | **71** | **64** | **65** | **65** | **65** | **64** | **71** | **71** | **71** | **71** | **66** | **66** | **64** | **57** | **58** | **56** | **59** | **58** | **58** | **58** | **56** | **58** | **58** | **58** | **57** | **56** | **57** | **57** | **57** |

1, gene present; 0, gene absent; blue, reference strains

**Table S4:** Listeriolysin O amino acid sequence comparison among 25 *Listeria monocytogenes* isolated from surface water

| Amino acid position and substitution[1] | Type of amino acid substitution | Strains | Remarks |
|---|---|---|---|
| H31N | Non-conservative | L164,L52,L105, L41,L124,L12,L58, L49 | |
| V433I | Conservative | L188,L57,L111,L127 | CC412 only |
| S523K | Conservative | L28, L44, L42, L128-3, L174, L49, L58, L105, L124, L12, L164, L52, L41, L36 | |

[1] Amino acid positions are relative to *L. monocytogenes* EGDe LLO protein

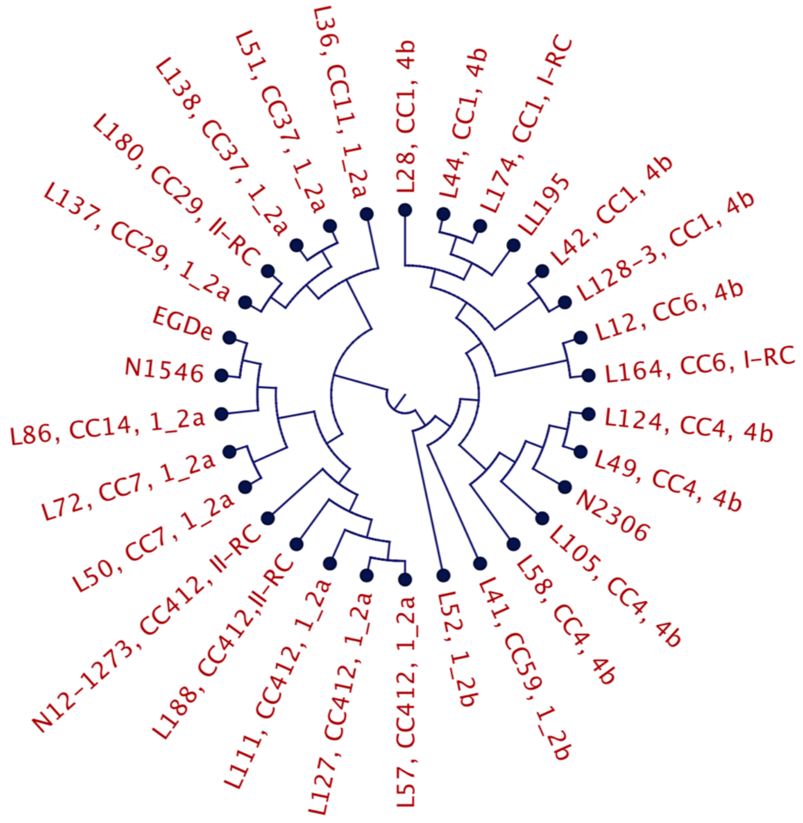


**Figure S1:** Phylogeny circular cladogram of the isolates based on average nucleotide identity (ANI) comparison showing the relationship of 25 *Listeria monocytogenes* isolated from surface water and five reference strains.

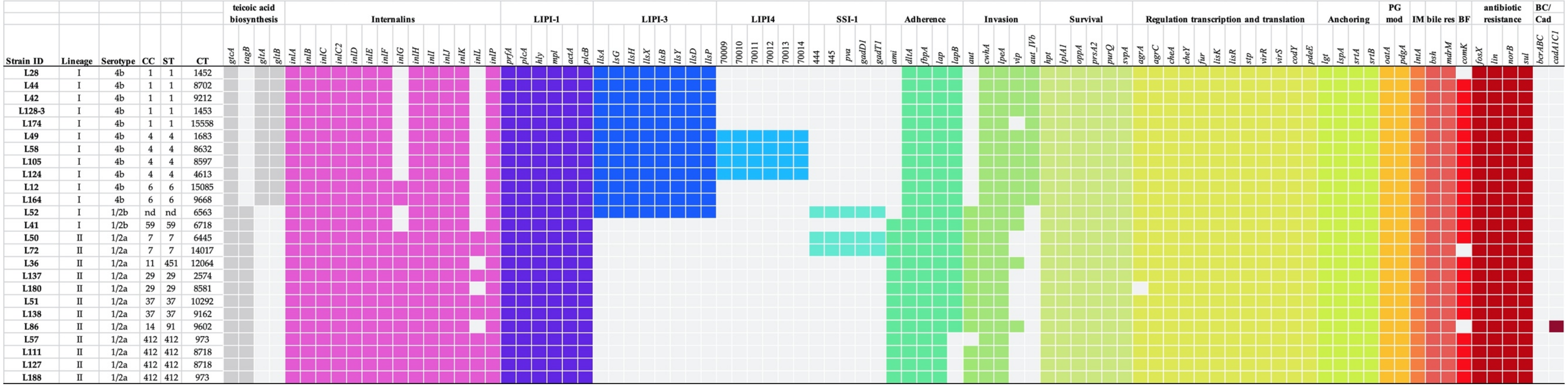


**Figure S2:** Virulence and resistance profiles of 25 *Listeria monocytogenes* isolated from surface water. Presence (color box) or absence (white box) of genes.
